# Hyperthermia elevates brain temperature and improves behavioural signs in animal models of autism spectrum disorder

**DOI:** 10.1186/s13229-023-00569-y

**Published:** 2023-11-15

**Authors:** Ana Belen Lopez-Rodriguez, Carol L. Murray, John Kealy, Clodagh Towns, Andrew Roche, Arshed Nazmi, Michelle Doran, John P. Lowry, Colm Cunningham

**Affiliations:** 1 School of Biochemistry and Immunology, Trinity Biomedical Sciences Institute and Trinity College Institute of Neuroscience, Trinity College Dublin, Dublin, Republic of Ireland; 2 Department of Chemistry, Maynooth University, Maynooth, Co. Kildare, Republic of Ireland

## Abstract

**Background:**

Autism spectrum disorders (ASD) are predominantly neurodevelopmental and largely genetically determined. However, there are human data supporting the idea that fever can improve symptoms in some individuals, but those data are limited and there are almost no data to support this from animal models. We aimed to test the hypothesis that elevated body temperature would improve function in two animal models of ASD.

**Methods:**

We used a 4 h whole-body hyperthermia (WBH) protocol and, separately, systemic inflammation induced by bacterial endotoxin (LPS) at 250 µg/kg, to dissociate temperature and inflammatory elements of fever in two ASD animal models: C58/J and Shank3B- mice. We used one- or two-way ANOVA and t-tests with normally distributed data and Kruskal–Wallis or Mann–Whitney with nonparametric data. Post hoc comparisons were made with a level of significance set at *p* < 0.05. For correlation analyses, data were adjusted by a linear regression model.

**Results:**

Only LPS induced inflammatory signatures in the brain while only WBH produced fever-range hyperthermia. WBH reduced repetitive behaviours and improved social interaction in C58/J mice and significantly reduced compulsive grooming in Shank3B- mice. LPS significantly suppressed most activities over 5–48 h.

**Limitations:**

We show behavioural, cellular and molecular changes, but provide no specific mechanistic explanation for the observed behavioural improvements.

**Conclusions:**

The data are the first, to our knowledge, to demonstrate that elevated body temperature can improve behavioural signs in 2 distinct ASD models. Given the developmental nature of ASD, evidence that symptoms may be improved by environmental perturbations indicates possibilities for improving function in these individuals. Since experimental hyperthermia in patients would carry significant risks, it is now essential to pursue molecular mechanisms through which hyperthermia might bring about the observed benefits.

**Supplementary Information:**

The online version contains supplementary material available at 10.1186/s13229-023-00569-y.

## Background

Autism is a spectrum of disorders (autism spectrum disorder, ASD) that are predominantly developmental in nature and largely genetically determined, but there is tentative evidence that environmental changes can impact on symptoms. Fever has long been reported to improve symptoms in individuals with autism. Although this is largely based on carers’ testimony that their children displayed remarkable improvements during febrile episodes, there are some data to support this, with up to 25% of patients displaying evidence of improvement [1]. That study described improvements in repetitive behaviours, stereotypy and irritability and some improvement in speech that coincided with fever of ≥ 100.4 °F (≥ 38 °C). More recent studies have added that carer-reported improvements occur predominantly in those with lower non-verbal cognitive skills and with more repetitive behaviours [2]. Importantly, given the neurodevelopmental nature of ASD, indications that symptoms can improve transiently under certain conditions imply that the circuits affected may possess the structural integrity to perform relatively normally under certain conditions. Understanding the conditions under which these circuits perform optimally offers hope of improving activities of everyday life for those with autism.

Arising from the observation that some individuals with autism function at a higher level than normal during fever, one might speculate that a higher body temperature favours improved circuit function. Increased temperature increases synaptic vesicle release in brain sections [3] and spike frequency in the songbird motor pathway [4] and cerebral metabolism has been shown to increase linearly with increased brain temperature [5]. In both rats and humans, glucose metabolism is altered by hyperthermia in a regionally heterogeneous manner [6]. However, during fever, such as that brought about by infection, there is significant production of inflammatory mediators and it is plausible that these mediators, rather than temperature change per se, may be the drivers of altered function in the central nervous system, as has been shown in a recent study implicating IL-17a in improving social behaviour in a maternal immune activation (MIA) model in the absence of significant hyperthermia. Since several pyrogenic pro-inflammatory mediators such as IL-1β, IL-6 and prostaglandins also suppress motivation, mood, social activity, arousal and cognition [7], it is even possible that the effects of fever might actually be more impressive if elevated temperature was achieved in the absence of inflammation. Despite the persistence of the fever hypothesis in the literature for many years, there are no credible behavioural neuroscience studies, demonstrating that fever-range temperature can improve function in animal models of ASD.

Bacterial endotoxin (lipopolysaccharide, LPS) is a pyrogen and can be used to mimic the acute phase of bacterial infection. However, LPS experiments must be performed at ambient temperatures that are thermoneutral for mice (i.e. 31 °C) in order to produce a 39 °C fever in C57BL6 mice [8]. Conversely, LPS typically produces either a mild hypothermia or limited change in body temperature at ambient temperatures of around 22 °C [9]. Therefore, one can use LPS at room temperature to induce systemic inflammation without marked elevation of body temperature. Conversely, whole-body hyperthermia (WBH) can be used to induce fever-range temperatures without triggering the acute inflammation occurring during LPS-induced inflammation [10]. Therefore, we can experimentally dissociate systemic inflammation from fever in order to compare cellular, molecular and behavioural effects of inflammatory and thermal stimuli.

Among the ASD symptoms reported to be alleviated during fever were traits such as hyperactivity, stereotypy and irritability, all of which could conceivably be suppressed as a result of lethargy and reduced locomotor activity that were likely to be present during the suppressive ‘sickness behaviour’ response of these subjects. Lethargy was described during fever in children with autism [1], and while suppression of those ‘hyperactive’ behaviours listed may appear, to carers, to be beneficial, it is important to establish whether such suppression constitutes a reversal of specific symptoms or simply a suppression of general activity. Therefore, it is important to assess whether fever can boost ‘negative’ features like impaired social approach and anxiety-driven hypoactivity as well as suppressing ‘positive’ features like hyperactivity, repetitive behaviour, excessive grooming and stereotypy.

In the current study, we dissociate fever from its normal inflammatory underpinning in normal mice and examine its impact on brain and body temperature and on neuronal activation in order to identify what is common among these perturbations and what is distinct. Based on suppression of most spontaneous behaviours with bacterial LPS, we specifically address the hypothesis that hyperthermia, in the absence of inflammatory stimulation, can reverse key deficits in two mouse models relevant to ASD: the C58/J mouse and Shank3B- mice.

## Material and methods

### Animals

Male mice from three different strains were used at 10 weeks old. For initial temperature experiments, C57 mice (C57BL/6 J (#000664)) were used for all the treatments, and for the assessment of ASD-like models, C58 (C58/J (#000669)) and Shank3B- (B6.129-Shank3 < tm2Gfng > /J; heterozygous (#017688)) were used. All three strains were from The Jackson Laboratory. Animals were housed in cages of four at 21 °C with a 12 h light/dark cycle. Food and water access was ad libitum. All animal experimentation was performed under licence granted by the Health Products Regulatory Authority, Ireland, with approval from the local ethical committee and in compliance with the Cruelty to Animals Act, 1876, and the European Community Directive, 86/609/EEC. Every effort was made to minimize stress to the animals.

### Subcutaneous temperature transponders implantation:

Subcutaneous temperature transponders 14 mm wide and 2 mm diameter (IPTT-300 (BMDS) were implanted following the manufacturer’s instructions, under light isoflurane anaesthesia, at least three days before being subjected to the WBH protocol. The correct functioning of the transponder was checked before and after the implantation. Temperature monitoring was taken with the IPTT-300 thermoreader (BMDS).

### Brain-implanted thermocouples and real-time temperature recordings:

For thermocouple recordings of brain temperature, mice (*n* = 6) underwent stereotaxic surgery to implant MBR-5 intracerebral guide cannulae (ID 457 μm, OD 635 μm; BASi Research Products, USA) into the striatum. Mice were anaesthetized with isofluorane (4% for induction, 1.5–3.0% for maintenance; IsoFlo®, Abbott, UK). The surgical site was shaved and disinfected, and lidocaine was administered subcutaneously for local anaesthesia. The skull was exposed, and three stainless steel support screws were implanted into the skull. A burr hole was made over the striatum, and the guide cannula was lowered into the caudate putamen (0.3 mm A/P; ± 2.0 mm M/L; 3.0 mm D/V), allowing room for the thermocouple to extend into the striatum once inserted into the guide cannula. The guide cannula was cemented into place (Dentalon® Plus, Heraeus-Kulzer, Germany), and once set, the scalp was sutured to close the wound. All animals were given saline (0.9%; 3 ml/kg body weight) and perioperative analgesia was provided (0.3 mg/kg body weight; Buprecare®, AnimalCare Ltd., UK) before animals were allowed to recover in an incubator set to 28 °C, with access to a food gel and hydrogel. Following 7 days of recovery, mice underwent three days of brain temperature recordings in an animal recovery chamber (Vet-Tech. model: HE010). There were 4-day rest periods between each recording day. On each recording day, the plug was removed from the implanted guide cannula and the thermocouple probe was inserted into the striatum. The thermocouple was an ultrafast T-type implantable device (IT-23; ADInstruments Ltd., UK) which was modified to fit into the implanted MBR-5 guide cannula. Briefly, a 2 cm length of deactivated fused silica tubing (ID 320 μm, OD 430 μm; Trajan Scientific Europe Ltd., UK) was cut and then carefully inserted under a microscope into a BR microdialysis probe head (BASi Research Products) so that it protruded 7 mm beyond the end of the probe head shaft. A small amount of glue (WEICON Epoxy Minute Adhesive) was then applied to the end of the shaft to fix the silica in place. After ca. 1 h, the thermocouple was inserted through a 1.8 cm length of PEEK tubing (ID 860 μm, OD 1270 μm; Plastics One, Roanoke, VA, USA) which was pushed well up the probe so that it was out of the way until later gluing. The thermocouple was then carefully inserted into the silica tubing under a microscope until it protruded ca. 1 cm. A small amount of epoxy glue was applied to the end of the silica and the thermocouple gently pulled back until it was 1 mm from the end of the silica as confirmed using a digital calliper. This was then left to dry for ca. 1 h before placing some epoxy glue around the silica at the top of the BR probe head. The PEEK tubing was then immediately pulled down and carefully inserted into the epoxy. Following overnight storage, the modified thermocouple was connected to a T-type Thermocouple Pod (ADInstruments Ltd.), which was in turn connected to the pod port of an e-Corder (eDAQ Pty Ltd, Australia), and the operational characteristics (temperature vs. voltage output) tested using the suppliers’ guidelines (ML312 T-type Pod Manual, AD Instruments Ltd.). This involved placing the thermocouple in a jacketed cell (ALS Ltd, IJ Cambria Scientific Ltd, Llanelli, UK) attached to a thermostatically controlled circulating water bath (Julabo Corio CD-BC4, Fisher Scientific, Dublin, Ireland) and recording the temperature vs. voltage output in Chart TM (Version 5, eDAQ Pty Ltd) at a sampling rate of 1 Hz. Raw temperature recordings in vivo were made in Chart TM (Version 5, eDAQ Pty Ltd, Australia) at a sampling rate of 10 Hz. On each recording day, baseline brain temperature recordings were made for 1 h. Following baseline recordings, the mice went through 3 interventions: (a) Day 1: room temperature protocol; (b) Day 5: whole-body hyperthermia (WBH) protocol; and (c) Day 9: LPS protocol (250 mg/Kg; i.p.). During brain temperature recording experiments, core body temperatures were measured using subcutaneous temperature transponders (IPTT-300 (BMDS) and the IPTT-300 thermoreader (BMDS) every 20 min without removing the mouse from the test chamber. All activity was marked on the real-time brain temperature recording.

### Whole-body hyperthermia and LPS treatment

**Fig. 1:**
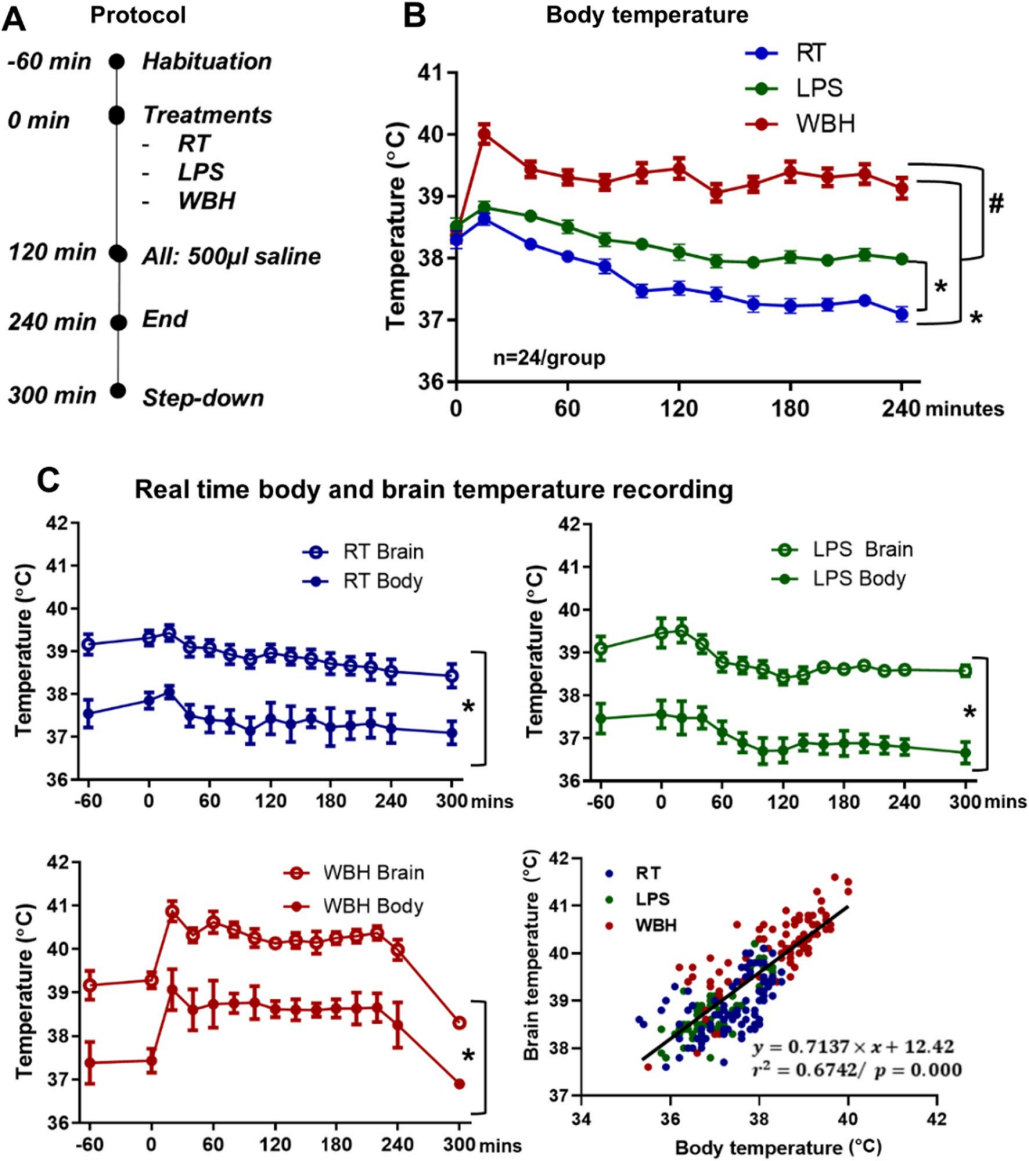
Whole-body hyperthermia produces elevation of body and brain temperature. **A** Schematic timeline of the whole-body hyperthermia (WBH) protocol, starting at the −60 min point for habituation, ending at 240 min of WBH treatment and refined by a step-down period to slowly cool down the animals up to 300 min. **B** Body temperature time course (°C) over the five hours duration of the protocol, measured by subcutaneous temperature transponders. Data are shown as Mean ± SEM (*n* = 24 for each group) and are analysed by repeated measures two-way ANOVA (* vs. RT group; # vs. LPS. Bonferroni post hoc, p < 0.05 °C). **C** Real-time body and brain temperature (°C) over the five hours duration of the protocol. Brain temperature was monitored using brain-implanted thermocouples in a small subset of those animals monitored by subcutaneous transponders (*n* = 5 for LPS and 6 for other groups). Abbreviations: RT room temperature; LPS intraperitoneally injected lipopolysaccharide, 250 µg/kg; WBH whole-body hyperthermia

Mice were exposed to whole-body hyperthermia (WBH) for 4 h by transferring them to a small animal recovery chamber (Vet-Tech. model: HE010) set at 38.5 °C (30 ± 1% humidity) to reach the target body temperature of 39.5 ± 0.5 °C. This was adapted for C58 strain (see results section). WBH protocol was invariably performed at the same time of day (8 a.m.) to minimize the effects of the circadian rhythm that occurs in the body temperature of rodents. Prior to starting the WBH protocol, the animals were transferred from their home cage to an empty new cage without bedding, food or water to allow them to habituate to a new space (T_−1 h_) and their body temperature and weight were taken. One hour later (T_0h_), their body temperature was recorded again using the thermoreader and subcutaneous temperature transponder (IPTT-300, BMDS) and they were injected with 500 µl either saline for control and WBH groups or LPS (250 µg/kg) before being transferred to the WBH chamber or the room temperature (RT) chamber (21 ± 1 °C; 50 ± 1% humidity). This LPS dose was chosen in order to ensure lethargy (as was observed in human studies of fever and ASD) but in the absence of robust hyper- or hypothermia (see Additional file 1: Fig. 2). Animals were left to freely move and explore, and their temperatures were taken every 20 min from T_0h_ to T_4h_. Two hours after starting WBH (T_2h_) the temperature and weight of the animals was taken and every animal, independent of treatment, was injected with 500 µl of saline to avoid dehydration and quickly returned to the chamber. Temperature recording every 20 min was resumed. At 4 h, temperature and weights were taken and every animal, independent of treatment, was again injected with 500 µl of saline. Although some WBH animals did have short episodes of jumping in the heating chamber, they were largely lethargic for the 4-h period and, on removal from the heating chamber, did rapidly re-establish normal locomotor behaviour, while LPS animals remained lethargic for many hours. For molecular experiments, animals were euthanized immediately after the WBH protocol and for behavioural studies, they underwent a ‘step-down’ cooling protocol to avoid the rebound hypothermia that occurs after WBH [11]. The ‘step-down’ protocol was as follows: the heated animals were returned to the WBH chamber, which was set, sequentially, at 32, 28, 23 °C and RT, for 20 min at each point in the sequence. Their temperature was also monitored, and after the step-down protocol, they were either returned to their home cage or started a panel of behavioural tasks (T_5h_), depending on the requirements of the experiment. (See Fig. 1 for protocol schematic).

### Tissue preparation

For the analyses of transcriptional changes, animals were terminally anaesthetized with sodium pentobarbital at 4 h post-WBH protocol (Euthatal; Merial Animal Health) and rapidly transcardially perfused with heparinized saline before the dissection of hypothalamus, hippocampus and amygdala that were snap frozen in liquid nitrogen and stored at − 80 °C until use. Animals for immunohistochemical examination were terminally anaesthetized with sodium pentobarbital (Euthatal; Merial Animal Health) and transcardially perfused with heparin–saline followed by 4% paraformaldehyde (PFA). Brains were gently removed and postfixed in 4% PFA. Coronal sections, 50 μm thick, were obtained using a Vibratome (Leica, Laboratory Instruments and Supplies, Ashbourne) to perform immunohistochemistry.

### RNA extraction, cDNA synthesis and quantitative PCR

Total RNA was isolated using the RNeasy Plus Mini method (Qiagen, Limburg, the Netherlands) following the manufacturer’s instructions. The RNA yield and quality of each sample were quantified based on optical density (OD) using the NanoDrop ND-1000 UV–Vis spectrophotometer (Thermo Fisher Scientific). cDNA synthesis was carried out using a high-capacity cDNA Reverse Transcriptase Kit (Applied Biosystems, Warrington, UK). Primer and probe sets were designed using NCBI Nucleotide tool and amplified a single sequence of the correct amplicon size, as verified by SDS-PAGE. Primer pair/probe sequences are shown in Table 1. Samples for RT-PCR were run in duplicate using FAM-labelled probes or SYBR green dsDNA-intercalating fluorescent dye (Roche) in a StepOne Real-Time PCR system (Applied Biosystems, Warrington, UK) under the cycling conditions: 95 °C for 10 min followed by 95 °C for 10 secs and 60 °C for 30 secs for 40–45 cycles. Quantification was achieved by exploiting the relative quantitation method. We used cDNA, prepared from isolated RNA that was pooled from the brains of WBH-treated and LPS-injected mice as a standard that expressed all genes of interest. Serial 1 in 4 dilutions of this cDNA were prepared in order to construct a linear standard curve relating cycle threshold (CT) values to relative concentrations, as previously described [12]. Gene expression data were normalized to the housekeeping gene 18S and expressed as relative concentration.

**Table 1:** Primer and probe sequences for quantitative PCR

Gene	Access. num	bp	Sequence
*Il1b*	M15131	69	Forward: 5'GCACACCCACCCTGCA3' Reverse: 5'ACCGCTTTTCCATCTTCTTCTT3' Probe: 5'TGGAGAGTCTGGATCCCAAGCAATACCC3'
*Il6*	NM_031168	72	Forward: 5'-TCCAGAAACCGCTATGAAGTTC-3' Reverse: 5'-CACCAGCATCAGTCCCAAGA-3' Probe: 5'-CTCTGCAAGAGACTTCCATCCAGTTGCC-3'
*Cox2*	NM_011198.4	81	Forward: 5’-TGGGTGTGAA GGGAAATAA GGA-3’ Reverse: 5’-GAAGTGCTGG GCAAAGAATG-3’
*Rankl*	NM_011613.3	112	Forward: 5'-GGGGGCCGTGCAGAAGGAAC-3' Reverse: 5'-CTCAGGCTTGCCTCGCTGGG-3'
*Igf1*	BC_012409	218	Forward: 5'-CTGGACCAGAGACCCTTTGC-3' Reverse: 5'-AGAGCGGGCTGCTTTTGTAG-3'
*Oxt*	NM_011025.4	376	Forward primer: 5’-TTG CTG CCT GCT TGG CTT AC-3’ Reverse primer: 5’-TAT TCC CAG AAA GTG GGC TC-3’
*Avp*	M88354.1	141	Forward primer: 5’-GCTGCCAGGAGGAGAACTAC-3’ Reverse primer: 5’-AAAAACCGTCGTGGCACTCG-3’

All probes used FAM as reporter. Where probe sequence is not shown, these assays were performed using SYBR green

### Immunohistochemistry

Immunohistochemistry was carried out on free-floating sections under moderate shaking. All washes and incubations were done in 0.1 M phosphate buffer pH 7.4, containing 0.3% bovine serum albumin and 0.3% Triton X-100. The endogenous peroxidase activity was quenched in a solution of 3% hydrogen peroxide in 30% methanol. Sections were incubated overnight at 4 °C with anti-c-Fos (C-10) mouse monoclonal IgG2 (Santa Cruz Biotechnology, Heidelberg, Germany), diluted 1:1000 in the presence of 5% normal horse serum (Vector Laboratories Inc., Burlingame, CA). Next day, sections were incubated for 2 h with biotinylated horse anti-mouse secondary antibody (1:300, Vector). After several washes in phosphate-buffered saline (PBS), ABC method was used (Vectastain, PK6100, Vector) and the reaction product was revealed using 3, 3’ diaminobenzidine as chromogen (Sigma-Aldrich) and H_2_O_2_ as substrate. Finally, sections were dehydrated, mounted on gelatinized slides, coverslipped and photographed using an Olympus DP25 camera (Mason) mounted on a Leica DM3000 microscope (Laboratory Instruments and Supplies, Ashbourne), captured using CellA™ software (Olympus, Mason).

### cFos analyses

The assessment of cFos activation was by a qualitative analysis using series of sections separated by 300 µm, beginning from the olfactory bulbs and continuing to the brain stem. Microscope images were taken at 10 × and 20x (*n* = 5–6). Regions of interest were selected based on prior studies used to capture the two main components of fever: temperature and inflammation. Thus, regions were chosen based on previous works that analysed cFos activation under cold/warm temperature protocols and after LPS treatment [13–15]. However, we also performed an anterior to posterior screen to positively identify any brain regions that showed particularly robust activation with respect to RT controls. Therefore, selection was not performed blind to treatment. Images for brain regions of interest, at positions along the anterior–posterior axis (with reference to the Allen Brain Atlas), were taken at either 10 × or 5 × magnification (*n* = 5–8), and for each photographed region, all sections were captured under the same conditions of light intensity, exposure, colour balance and saturation. Labelled cFos cells were analysed using Fiji (an open-source image processing package built off ImageJ2 software). Images were converted to 8 bit and thresholded before setting particle size (> 50) and circularity values (0.5–1). Throughout this process, cells detected by ImageJ automatic counts were compared visually to the original photograph and sample manual quantitative counts of cFos cells were compared to automatic counts to verify the validity of the methodology. Cell counts are expressed as cells/mm^2^.

### Blood glucose measurements

Two different methods were used to assess blood glucose. For serial sampling, the blood glucose levels were measured via serial tail vein microsampling (less than 10 μl) one hour before starting the protocol and then at 40 min, 2 h, 4 h, 7 h and 24 h. Animals were bled using a 30G lancet, and glucose was determined in the blood drop with the precision Xtra glucometer (Abbott). In a different set of experiments, the glucose levels were determined in the first drop of blood from the right atrium immediately before performing transcardial perfusion (4 h or 24 h after the heating protocol).

### Plasma ELISA assays

Animals were terminally anaesthetized at 4 h post-WBH protocol with sodium pentobarbital (Euthatal, Merial Animal Health). The thoracic cavity was opened, and blood was collected in heparinized tubes directly from the right atrium of the heart. This whole blood was spun at 1.5 × g for 15 min to remove cells; the plasma was then aliquoted and stored at − 20 °C until use. These samples were then diluted appropriately and analysed for IL-1β, TNF-α and IL-6 by sandwich-type ELISA, using ELISA MAX Mouse IL-6, ELISA MAX Mouse IL-1β (Biolegend, San Diego, USA) and DuoSet Mouse TNF-α (R&D Systems, Minneapolis, USA). The required capture and detection antibodies, cytokine standard and avidin–HRP (IL-6) or streptavidin–HRP (TNF-α) were supplied with each respective kit; however, for IL-1β a more sensitive streptavidin poly-HRP (Sanquin, Amsterdam, the Netherlands) was used in place of the supplied one. Optical density was read at 450 nm with correction at 570 nm. Standard curves for each antibody were used, and samples were quantified only if the absorbance fell on the linear portion of the standard curve. Reliable quantification limits for the assays used were IL-1β 31.25 pg/ml, TNF-α 15.6 pg/ml and IL-6 15.6 pg/ml.

### Experimental design of behavioural studies

Given the heating protocol, it was necessary to test several behaviours in a short time after the animal had emerged from the heating protocol. Therefore, we prioritized those behaviours that were most important to assess in these distinct strains. The criteria for prioritizing individual tasks for each strain were: 1) That those behavioural indices were demonstrably altered (at baseline) in the ASD strain of choice with respect to normal controls. 2) That the assembled test battery contained tasks that could be regarded as ‘positive symptoms’, i.e. higher scores are present in the ASD strain with respect to controls, and ‘negative symptoms’, i.e. lower scores are present in the ASD strain with respect to controls. This was done so that ‘improvements’ in these ASD-relevant behaviours could not be confounded by a general suppression of all spontaneous activity. Therefore, careful phenotyping was essential at baseline, even though assessment of those was not novel per se*,* in order to develop test batteries that allowed us to select for tasks that showed impairments in our chosen strains, in our hands (see Figs. 6 and 8). From those baseline measures, we then selected the most sensitive battery of tests that could be conducted in a short time frame after ASD strains were removed from the heating chamber. Importantly, the selected behavioural tasks included behaviours that are well known as hallmarks of these strains, but not all tasks listed below could be performed in WBH experiments with each strain. Experiments were performed using independent cohorts of mice since strains were obtained at different times and because the size of cohorts that were required to perform these analyses could not accommodate experiments with > 2 strains. In the first instance, C57 mice were used to illustrate that WBH and LPS had quite distinct effects on spontaneous behaviour in the hours after treatment and independent cohorts of C57 mice were used subsequently when acting as controls in experiments with C58 and Shank3B mice since each of these experiments had to be conducted and presented independently.

### Burrowing

Burrowing is a species typical behaviour and was performed following Deacon’s protocol [16, 17]. Burrows were made from a 200-mm-long, 68-mm-diameter black plastic tube. One end of the tube was closed with a piece of plastic from the same material, and the other end was open and raised 30 mm above the cage floor by two 50 mm screws. Animals were placed into individual opaque cages with fresh bedding and provided with water and the burrowing tube filled with 300 g of food pellets as substrate. The food pellets remaining in the burrowing tubes were measured after 2 h and at 24 h, and this weight was subtracted from 300 g to calculate the burrowing activity for each mouse. At 24 h, the mice were returned to their home cages.

### Open-field activity

The open-field test was used to assess spontaneous activity in a novel environment, and it also served as the habituation period for the social interaction test that immediately followed it (detailed below). Briefly, mice were allowed to freely and individually explore an open-field arena (58 × 33 × 19 cm; divided into squares of 10 × 10 cm) for 10 min. Activity was monitored via an overhead camera and recorded using AnyMaze software (version 4.99). The mice were assessed on parameters such as distance travelled, mean speed, rotations, time spent in the outer and inner zone and time freezing.

### Social interaction

We assessed social interaction using a rectangular arena 58 × 33 × 19 cm. Two wire mesh cages (9 cm diameter) were placed in the middle of each half of the arena. The test began with a habituation period to the arena for 10 min. (This was used to take measurements of locomotor activity as described in ‘Open-field activity’.) Thereafter the mouse was allowed to adapt, for 5 min to the placement of two small empty wire mesh cages for 5 min. Finally, the social preference test was conducted for 10 min, during which an unfamiliar (stranger) conspecific mouse of the same age, weight and sex was placed into one of the mesh cages, whereas the other mesh cage contained an inanimate object. AnyMaze software was linked via an overhead camera and used to recorded test mice, tracking the movement of the test mice, and recording parameters such as time spent sniffing the novel mouse and the number of sniffs, time spent in the area of the mouse mesh cage vs. the empty cage and time freezing. After the trial, the arena and wire cages were cleaned with 10% ethanol.

### Backflips and upright scrabbles

Backflips and upright scrabbles have been described as specific behavioural alterations of the C58/J strain [18–22]. The test has two parts. First, a period (5 min) of adaptation to an opaque plastic cage (19.5 × 31 × 13 cm) with a regular metal bar lid placed on top provided with fresh bedding for each mouse. The second part consisted of observation and counting of the number of backflips and upright scrabbles for a period of 30 min. Back flipping manifests as backward somersaulting, often with the assistance of the cage lid, while upright scrabbling consisted of rapidly running or climbing ‘on the spot’, usually against a wall or corner, which may be related to wall-climbing stereotypy. Neither of these stereotypies were ever observed in C57 or Shank3B mice.

### Marble burying

An opaque plastic cage (45 × 23 × 13 cm) was used for this test. The cage was filled approximately 6–8 cm deep with wood chip bedding, lightly patted down to flatten the surface and make it even. A regular pattern of 20 glass marbles was placed on the surface: 5 columns 8 cm apart and 4 rows 4 cm apart. The mouse was placed in the cage and left for 30 min. After that time, the marbles that were completely buried or buried to 2/3 their depths were counted.

### Grooming

One of the specific behavioural alterations of the Shank3B- strain is excessive, sometimes injurious grooming [23–25]. To assess this, mice were placed individually in a clean clear plastic cage with fresh bedding and the lid, and they were left for 5 min to adapt to the new environment. After that, the time spent grooming was measured over 5 min.

### Elevated zero maze

Shank3B- mice have been shown to spend less time in the open arms during the Elevated-Zero-Maze (EZM) [24, 25]. The EZM is a modification of the plus-maze based on two conflicting innate tendencies: exploring a novel environment and avoiding elevated and open spaces that constitute a risky situation. The EZM consists of an elevated circular track divided into 4 equal lengths: 2 lengths of track enclosed by an opaque wall on both sides and 2 equal lengths that do not have a surrounding wall. Open and closed areas alternate. Mice were placed individually into one of the closed arms and left to explore for 5 min. The time spent in exploring enclosed versus open arms, the latency to enter the open arms and the number of risk assessment events were counted.

### Horizontal bar

Shank3B- mice present mild motor abnormalities that are increased in Shank3KO [24–26]. The horizontal bar test was used to assess the muscular strength, motor coordination and prehensile reflex. This test consisted of a 26-cm-long, 0.2-cm-diameter metal bar, supported by a 19.5-cm-high column at each end. Each mouse was held by the tail, placed with its front paws at the central point of the bar and rapidly released. A score was assigned depending on whether and when the mouse fell, whether it held on for 60 s or whether it reached a supporting column. Animals score 1 if they fell off within 10 s, score 2 if they held on for 11–59 s, score 3 if they held on for 60 s or reached the safe platform in 60 s, score 4 if they reached the safe platform within 30 s and score 5 if they reached the platform within 10 s.

### Statistical analyses

All statistical tests employed, and results obtained are compiled in Additional file 1: table 1. For two-group comparisons, data were analysed using unpaired two-tailed t-test when they were normally distributed and the Mann–Whitney test if data did not pass the assumptions for parametric analyses. For correlation analyses, data from all groups were pooled and adjusted by a linear regression model. One-way analysis of variance (ANOVA) was performed to compare RT, LPS and WBH on molecular changes and on cFos positive cell counts. Multiple groups were also analysed by repeated measures, two-way, analysis of variance (ANOVA), with treatment (room temperature (RT), LPS or whole-body hyperthermia (WBH) and time (0–48 h) as independent factors. Post hoc comparisons (Bonferroni's test or Fishers LSD where correction for multiple comparisons was not required) were made with a level of significance set at *p* < 0.05. Data from 2 factor experiments were not always normally distributed, and in these cases, nonparametric tests were used (Kruskal–Wallis and post hoc pairwise comparisons with Mann–Whitney U-test). Post hoc comparisons were made with a level of significance set at *p* < 0.05. Data are presented as mean ± standard error of the mean (SEM) or median ± interquartile range where nonparametric. Symbols in the graphs denote post hoc tests. Statistical analyses were carried out with the SPSS 22.0 software package (SPSS, Inc., Chicago, IL, USA).

## Results

### Whole-body hyperthermia protocol characterization

The experimental protocol is shown in Fig. 1A. The whole-body hyperthermia (WBH) protocol induced an increase in the body temperature reaching the target body temperature of 39.5 ± 0.5 °C (fever-like body temperature). Neither LPS-treated animals (250 μg/kg i.p.) nor RT animals showed a sustained increase in temperature from baseline, although LPS-treated remained at a significantly higher body temperature than RT mice (Fig. 1B). Repeated measures two-way ANOVA showed a significant effect of treatment [*F*_2,69_ = 78.6, *p* < 0.0001], of time [*F*_12,828_ = 40.61, *p* < 0.0001] and an interaction between these two factors [*F*_24,828_ = 13.19, *p* < 0.0001]. Subsequent Bonferroni’s multiple comparison test indicated a significant difference between RT and WBH, between WBH and LPS and between RT and LPS (all *p* < 0.0001). Body and brain temperature were also monitored simultaneously using subcutaneous transponders and brain-implanted thermocouples over the four hours duration of the WBH protocol (*n* = 6 per group except *n* = 5 for LPS) (Fig. 1C). Brain temperature was consistently significantly higher than body temperature, in all 3 treatment groups. There was a significant effect of body region (i.e. brain vs. whole body) when data were analysed by two-way ANOVA repeated measures in RT [*F*_1,5_ = 43.12; *p* = 0.0012], LPS [*F*_1,4_ = 197.1; *p* = 0.0001] and WBH [*F*_1,5_ = 65.27; *p* = 0.0005] treated animals. The WBH protocol produces an acute elevation in brain temperature that persists as long as the WBH protocol continues, and returns to baseline temperature thereafter, just as it does in the body as a whole. When all three groups were pooled and analysed by linear regression, there was a statistically significant and strong positive correlation between body and brain temperature (*r*^2^ = 0.6742) and this persists even when body temperature is elevated, as in the case of the WBH animals. For experiments in which subsequent behavioural assessment was necessary, the WBH protocol had to be refined by including an extra ‘step-down’ period (as per methods section) to allow animals to cool gradually in order to avoid rebound hypothermia [11] (Fig. 1A).

### Inflammatory markers induced by LPS and WBH

**Fig. 2:**
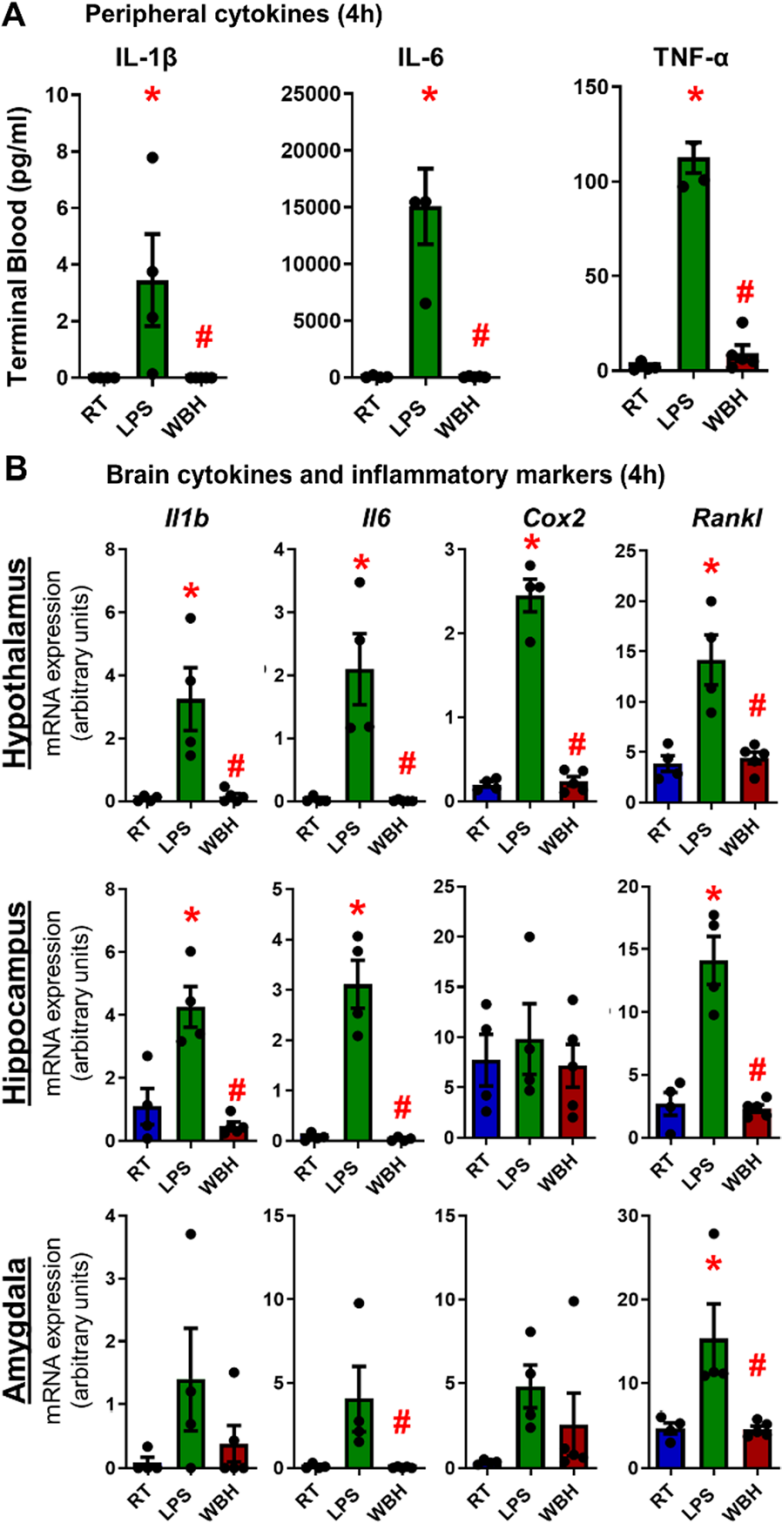
Inflammatory markers at 4 h post-whole-body hyperthermia (WBH) or LPS. **A** Peripheral blood cytokines measured at 4 h after the WBH protocol: IL-1β, IL-6 and TNF-α levels (pg/ml). **B** mRNA expression of brain cytokines and inflammatory markers in brain homogenates of hypothalamus, hippocampus and amygdala. Data are shown as mean ± SEM, with each data point representing one animal (*n* = 5 for WBH and *n* = 4 for LPS, RT). Data have been analysed by parametric or nonparametric statistics as appropriate to the data distribution: Kruskal–Wallis or one-way ANOVA followed by Mann–Whitney or Bonferroni’s tests p < 0.05. (* LPS vs. RT; # WBH vs. LPS). Abbreviations: RT room temperature; LPS intraperitoneally injected lipopolysaccharide, 250 µg/kg; WBH whole-body hyperthermia

Since fever is typically underpinned by an acute inflammatory response, we sought to ascertain whether raised body temperature per se induced increased peripheral or central inflammation comparable to inflammation induced by LPS. Plasma cytokine levels, assessed by ELISA, showed a clear induction of IL-1β, IL-6 and TNF-α in LPS-treated animals, but not in WBH animals (Fig. 2A). Kruskal–Wallis analysis revealed a significant effect of treatment on IL-1β, IL-6 and TNF-a levels [*F*_2,12_ = 9.750; *p* = 0.004] with LPS significantly different to both RT and WBH in all cases, while WBH was not different to RT in any case. The results indicate that LPS significantly increases circulating inflammatory cytokines, whereas WBH did not. Similar results were found in brain homogenates in three different regions in which *Il1b*, *Il6*, *Cox2* and *Rankl* mRNA levels were elevated by LPS treatment when compared to RT and WBH; however, WBH did not induce a brain inflammatory response and was not significantly different to RT controls on any gene in any region (Fig. 2B). Conversely, LPS was significantly different both from RT control and WBH animals. *Rankl* was significantly induced by LPS in all regions examined, while *Il1b* and *Il6* were induced in the hypothalamus and hippocampus, but were not significant in the amygdala owing to substantial variability in this region. *Cox2* was robustly elevated in the hypothalamus, but not in the hippocampus (high basal expression of *Cox2*) or in the amygdala (high variability). These experiments were powered based on the ability of LPS to produce robust levels of inflammatory transcripts and proteins [9, 27]. WBH does not induce significant inflammation, although the small number of subjects constrains interpretations in the amygdala. Additional file 1: table 1 shows full statistical design. Transcripts for heat shock proteins (HSPs; *Hspb1*, *Hsp105b* and *Hspa1a*) were assessed in hypothalamus, hippocampus and amygdala and statistically significant inductions were observed selectively with WBH treatment (Additional file 1: Fig. 1). The level of induction was modest and not all transcripts were induced in all regions.

### cFos activation

**Fig. 3:**
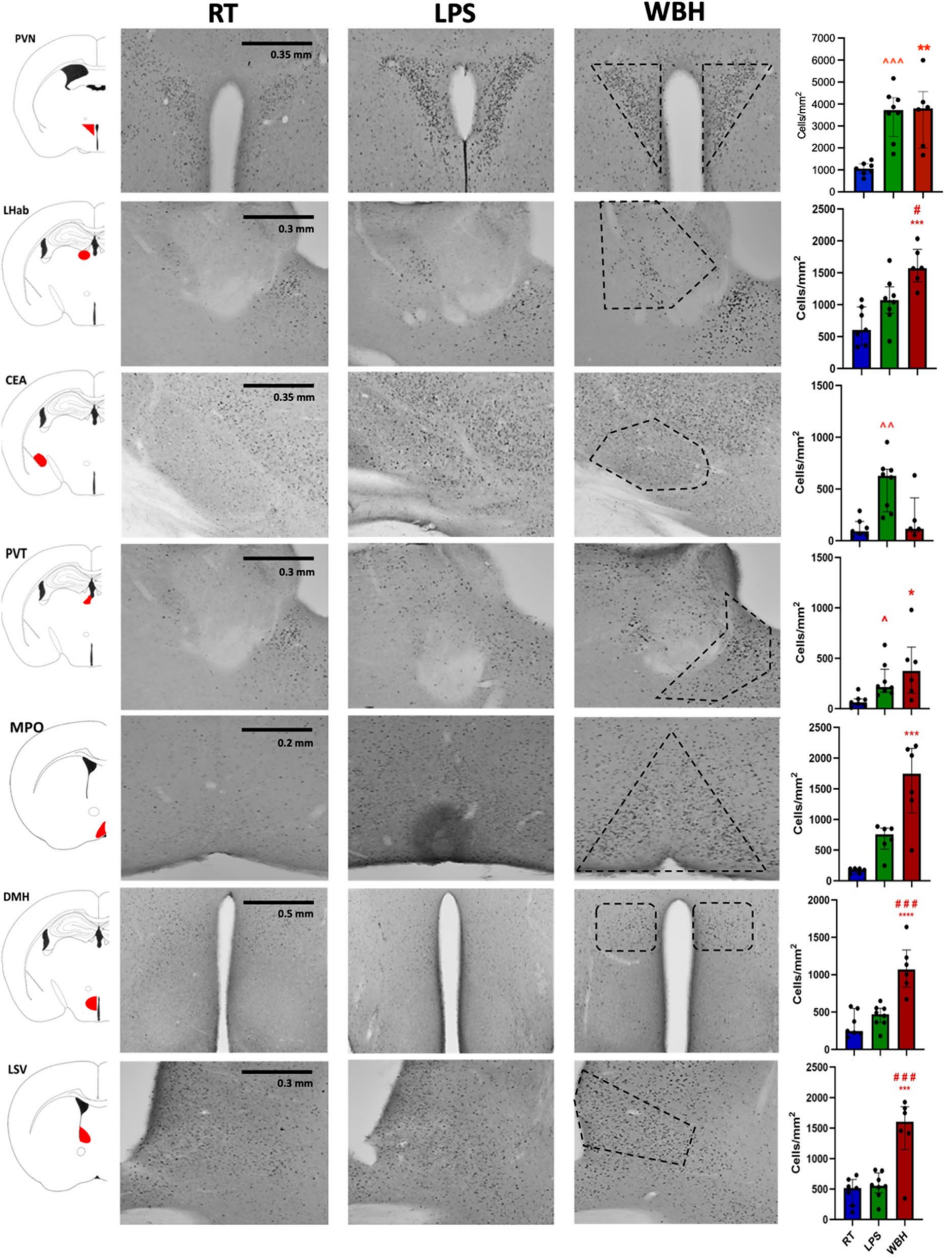
Differential patterns of labelling with cFOS. Immunohistochemistry for cFOS was performed in free-floating sections from brains of mice exposed to room temperature (RT), LPS (250 µg/kg) or whole-body hyperthermia (WBH) and quantitatively assessed (RT *n* = 7; LPS *n* = 8; WBH *n* = 6). Regions of interest are defined by their position from Bregma on the anterior–posterior axis. Abbreviations: PVN: paraventricular nucleus (− 0.58 to − 0.94 mm), LHab: lateral habenula (− 1.58 to − 2.06 mm), CeA: the central nucleus of the amygdala (− 1.58 to − 1.94 mm), PVT: paraventricular thalamus (− 1.46 to − 2.06 mm) MPO: medial preoptic nucleus (0.14–0.5 mm), DMH: dorsomedial hypothalamic nucleus (− 1.34 to − 1.94 mm) and LSV: lateral septum, ventral portion (0.3–0.76 mm). cFOS positive neurons were counted in the regions of interest, which are defined by the shapes indicated. Data are represented as mean ± SEM and analysed by one-way ANOVA where normally distributed and by median ± IQR and Kruskal–Wallis analysis when not normally distributed. After significant group analysis pairwise comparisons between LPS and RT are indicated by ^, between WBH and RT by * and between WBH and LPS by #. For all 3 symbols p values of 0.05, 0.01, 0.001 and 0.0001 are denoted by 1, 2, 3 or 4 repeats, respectively, of these symbols

Previous studies have shown that LPS treatment or environmental warmth (37 °C) induce neuron activation following a differential brain region pattern [13, 14]. To characterize differential neuron activation patterns, cFos immunostaining was performed and analysed at 4 h after WBH or LPS treatment. This approach explicitly did not seek to identify or quantify a specific locus for any particular behavioural effect. Rather this is a largely unbiased screen for major changes in neuronal activation that overlap or differ between WBH and LPS. cFos showed different activation levels after WBH or LPS treatment in a brain region-dependent manner. Although saline injection may produce some cFos activation, all treatment groups received i.p. saline at both 4 h and 2 h before euthanization so patterns of selective labelling reported here are distinct from any pattern that might be induced by saline injections. WBH tended to induce cFos activation that was higher, and present in more brain regions, than LPS treatment did (Fig. 3). Numbers of cFos positive cells in several exemplar regions showed significant group differences by one-way ANOVA (*F*_2,18_ ≥ 12.44, *p* ≤ 0.0017) or, when not normally distributed, by Kruskal–Wallis analysis (KW statistic ≥ 10.01, *p* ≤ 0.0029) in the paraventricular nucleus (PVN), lateral habenula (LHab), the central nucleus of the amygdala (CeA), paraventricular thalamus (PVT) medial preoptic nucleus (MnPO), the dorsomedial hypothalamic nucleus (DMH) and the lateral septum, ventral portion (LSV). LPS activated cFOS in the PVN, PVT, MPO and, uniquely, the CeA while WBH shared this pattern in PVN and PVT, but was more robust than LPS in activating the LHab, MPO, DMH, LSV. Pairwise comparisons are shown in Fig. 3 legend and in Additional file 1: table 1.

### Glucose levels and hypothalamic hormone transcripts:

**Fig. 4:**
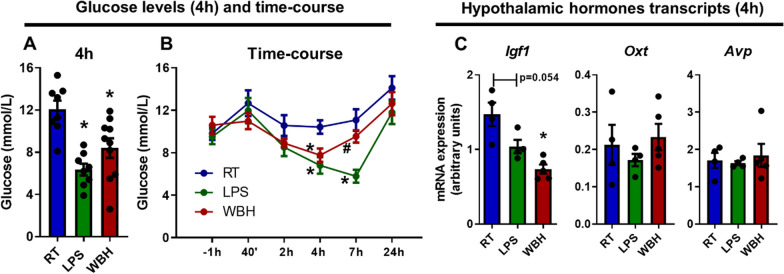
Glucose and hypothalamic hormone mRNA levels after LPS and WBH protocols. **A** Blood glucose levels (mmol/L) at 4 h after LPS or WBH, shown as mean ± SEM (RT, LPS *n* = 8, WBH *n* = 10). **B** Blood glucose levels (mmol/L) measured by serial tail vein sampling from 1 h pre- to 24 h post-LPS or WBH treatment (*n* = 8). **C** Level of expression of mRNA for hypothalamic hormones at 4 h after LPS or WBH. Data are shown as mean ± SEM, with each data point representing one animal. Time course analysis was performed using two-way repeated measures ANOVA followed by Bonferroni post hoc analyses. All other analyses performed by one-way ANOVA followed Bonferroni’s test. * versus RT; # versus LPS. Abbreviations: RT, room temperature; LPS, bacterial endotoxin, 250 µg/kg; WBH, whole-body hyperthermia

Significant metabolic changes are required to raise body temperature [28] and also occur during the response to LPS [29]. Here, both LPS and WBH had a significant impact on plasma glucose levels when compared with control (RT) animals (Fig. 4A). This was apparent in terminal bloods (4 h post-LPS or WBH) and, when using serial tail vein sampling, could be shown to emerge as soon as 2 h postchallenge (Fig. 4B) and to become statistically significantly different from RT in both groups by 4 h (main effect of treatment: *F*_2,23_ = 11.89, *p* = 0.0003). The decrease was greater in LPS-treated mice than in WBH, remaining significantly different to RT and WBH at 7 h (*p* = 0.0037 and 0.0017, respectively) (Fig. 4B). Effects of hyperthermia or LPS on mRNA levels were assessed for three hypothalamic-expressed hormones, all previously linked to ASD [30, 31] (Fig. 4C). One-way ANOVA showed a significant decrease in  *Igf1* mRNA (main effect of treatment: *F*_2,10_ = 12.78; *p* = 0.0018). Post hoc tests showed a significant decrease in *Igf1* only with WBH (*p* = 0.0015). No significant differences were observed for *Oxt* or *Avp*.

### Effects of WBH and LPS in C57 mice

**Fig. 5:**
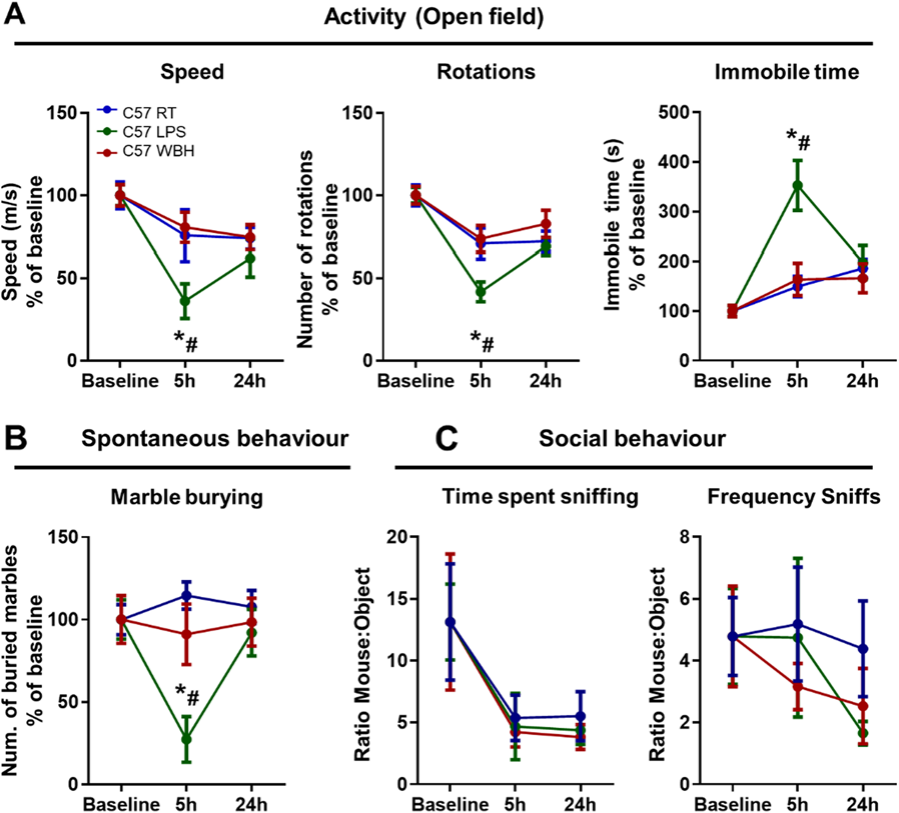
Effects of WBH and LPS in C57 strain. **A** Description of the effects of WBH and LPS on the general activity using the open-field test, showing the mean speed (m/s), number of rotations and immobility time (s) for 10 min. **B** Analyses of WBH and LPS effects on the number of marbles buried within 20 min. **C** Characterization of the effects of WBH and LPS on social behaviour using the time of sniffing and the frequency of sniffs of a novel mouse in comparison with an inanimate object (ratio mouse:object) for 10 min. Mean ± SEM (RT *n* = 12; LPS *n* = 8; WBH *n* = 12). Repeated measures two-way ANOVA. * vs. RT and # versus WBH by Bonferroni post hoc tests. (RT: room temperature; LPS: intraperitoneally injected LPS, 250 µg/kg; WBH: whole-body hyperthermia)

Bearing in mind the cellular and molecular changes observed in WBH and LPS mice, we assessed the impact of these stimuli on behaviour in normal C57 mice. Whole-body hyperthermia did not significantly affect the normal behaviour of C57 mice, whereas LPS induced a general decrease in activity as assessed in the open-field test (Fig. 5A). For the mean speed measurement, repeated measures two-way ANOVA showed a significant effect of treatment (*F*_2,29_ = 4.121; *p* = 0.027) and time (*F*_2,58_ = 9.898; *p* < 0.001) with Bonferroni post hoc test showing the LPS-induced suppression of spontaneous behaviour at 5 h compared with RT group (*p* = 0.021) and WBH (*p* = 0.008). Similar results were obtained for the number of rotations and reciprocal observations were made for the time spent immobile, showing that LPS significantly increased the time immobile at 5 h when compared with the RT group (*p* < 0.0001) and WBH (*p* < 0.0001). Marble burying also demonstrated a suppression of this behaviour by LPS, while there were no effects of WBH. There was a significant effect of treatment (*F*_2,29_ = 4.184; *p* = 0.025), which was driven by a significant effect of LPS at 5 h, in comparison with RT group (*p* < 0.0001) and WBH (*p* = 0.0051). In the social interaction tests, all animals showed suppression of social behavioural preference at 5 and 24 h, but there were no significant differences between RT, WBH and LPS (Fig. 5C).

Since LPS suppressed these spontaneous behaviours in C57 mice, we proposed that they would do likewise in C58 and Shank3B mice. Acute suppression of spontaneous activity was clear in all 3 strains (Additional file 1: Fig. 2), from which we infer typical LPS-induced inflammatory responses in these strains. Therefore, LPS was not pursued further as a suitable stimulus to examine acute improvements in function in ASD mouse models.

### Behavioural characterization of the C58 strain at baseline

**Fig. 6:**
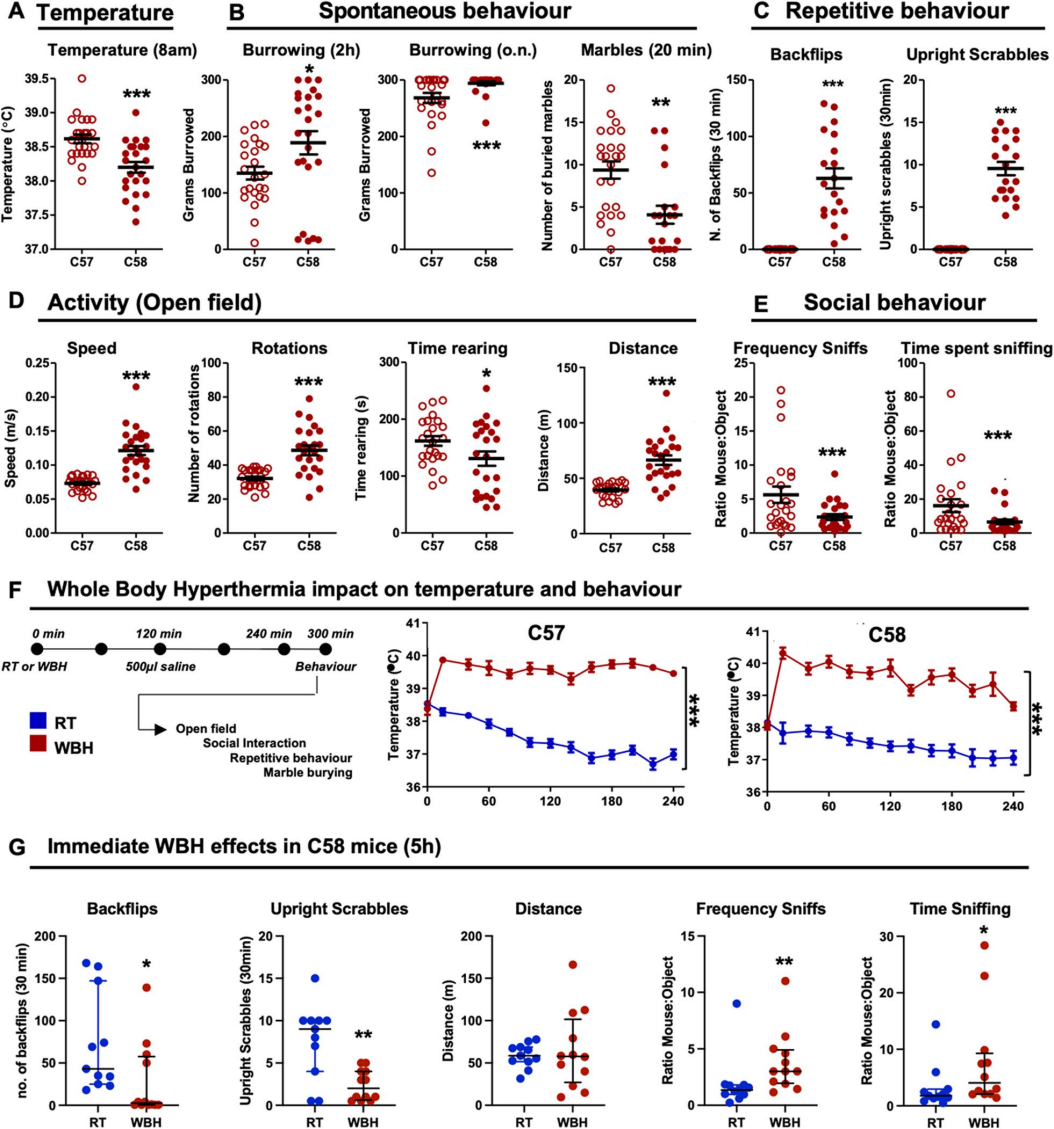
Characterization of basal behaviour in C58 mice. **A** Basal body temperature at 8am, as measured by subcutaneously implanted transponder (°C). **B** Spontaneous behaviour assessed by the amount of burrowed food pellets at 2 h, and overnight, and by marble burying for 20 min. **C** Description of the repetitive behaviours found in C58 strain: backflips and upright scrabbles for 30 min. **D** Analyses of the general activity using the open-field test, showing the mean speed (m/s), number of rotations, time rearing (s) and travelled distance (m) for 10 min. **E** Social behaviour characterization using the frequency of sniffs and the time of sniffing of a mouse in comparison with an inanimate object (ratio mouse:object) for 10 min. **F** Experimental scheme for assessment of the effect of WBH on behavioural parameters in C58 mice. **G** Impact of WBH on 5 behavioural indices, measured at 5 h after the beginning of WBH. Each animal is represented by a single data point and mean ± SEM are also indicated (*n* = 24 for each strain in (**A**–**E**) and 11–12 in (**G**). Data have been analysed by parametric or nonparametric statistics as appropriate to the data distribution: unpaired two-tailed t-test or Mann–Whitney, *, ** and *** denote p < 0.05, p < 0.01 and p < 0.001 in pairwise comparisons

The natural mutant C58 mouse was chosen as a suitable strain in which to assess the impact of WBH on autism-like features because they are reported to present several negative and positive symptoms/signs including modified spontaneous behaviours, stereotypy and highly characteristic repetitive movements like backflips and upright scrabbles, as well as decreased social interaction [18–22]. Since the purpose of this study was to examine WBH- and LPS-induced deviations from baseline behaviour in this and other ASD strains [32], it was first necessary to identify which of those previously reported behavioural signs in C58 mice, when compared to C57 mice, were most robust and suitable for repeat testing in subsequent experiments. Basal body temperature was measured at 8 am, before assigning to treatment groups (Fig. 6A) and C58 mice were found to have a significantly lower basal body temperature than C57 mice (*p* = 0.0001, unpaired two-tailed t-test, *n* = 24 per strain). Although some variability exists in C57 body temperatures across the study as a whole (see Additional file 1: Fig. 3), C57 body temperature distributions are not statistically different in Figs. 6 and 8. The C57 vs. C58 distributions are clearly different and all animals in Fig. 6A were measured together in order to minimize any influence of day-to-day variability. Burrowing of food pellets is a species typical ‘natural’ behaviour in mice [17, 33] and was significantly increased in C58 mice both at 2 h (*p* = 0.0273) and overnight (Mann–Whitney; *p* = 0.0041). Conversely, C58 mice showed significantly less marble burying (Mann–Whitney; *p* = 0.0012), which, thus, may described as a negative symptom in these mice. As in previous descriptions, we also observed a large number of backflips (Mann–Whitney; *p* < 0.0001) and upright scrabbles (Mann–Whitney; *p* < 0.0001) which were absent in C57 mice. These parameters may be regarded as ‘positive symptoms’. Hyperactivity has also been described in C58 mice and we used the open-field test to show a significant increase in the speed (*p* < 0.0001), number of rotations (Mann–Whitney; *p* < 0.0001) and travelled distance (*p* < 0.0001) and a modest but significant decrease in the time spent rearing (*p* = 0.0475) in C58 mice (Fig. 6D, Unpaired t-tests or Mann–Whitney). Finally, social behaviour was assessed using the social interaction test, indicating significant decreases in the frequency of (Mann–Whitney; *p* = 0.0196), and the time spent (Mann–Whitney; *p* = 0.0078), sniffing a novel mouse in comparison with an inanimate object (Fig. 6E). These provide robust indices with which to assess the impact of WBH on the behavioural parameters in the proceeding experiment shown in Fig. 6F, G.

### Immediate and persistent effects of WBH on C58 strain

The WBH protocol required adaptation to be used with C58 animals since the initial WBH protocol (38.5 °C) induced body temperature increases above 40 °C. In order to reach target body temperature of 39.5 ± 0.5 °C for the full 4 h (Fig. 6F C58: *p* < 0.0001, *F*_1,23_ = 139.2), the heating box was set at 33.5 °C when using the C58 strain. Immediately upon emergence from WBH both strains showed a short period of intense grooming, but this was a strain-independent feature and resolved relatively rapidly (Additional file 1: Fig. 4). WBH did not significantly affect any other assessed parameter in C57 control mice (Fig. 5 & Additional file 1: Fig. 2 ). When measured at 5 h, immediately upon mice returning to baseline temperature, WBH showed significant impacts on both backflips (*p* = 0.0145) and upright scrabbles (*p* = 0.0077), without any significant impact on locomotor activity per se (Fig. 6G, left). Marble burying was too variable at baseline, in both C57 and C58 mice, to perform statistical analysis so this was not pursued in WBH experiments. Despite suppression of repetitive behaviours, social interaction was improved by WBH, as measured by both frequency of sniffing (*p* = 0.0044) and the time spent sniffing (*p* = 0.0331) an unfamiliar conspecific mouse (Fig. 6G, right).

**Fig. 7:**
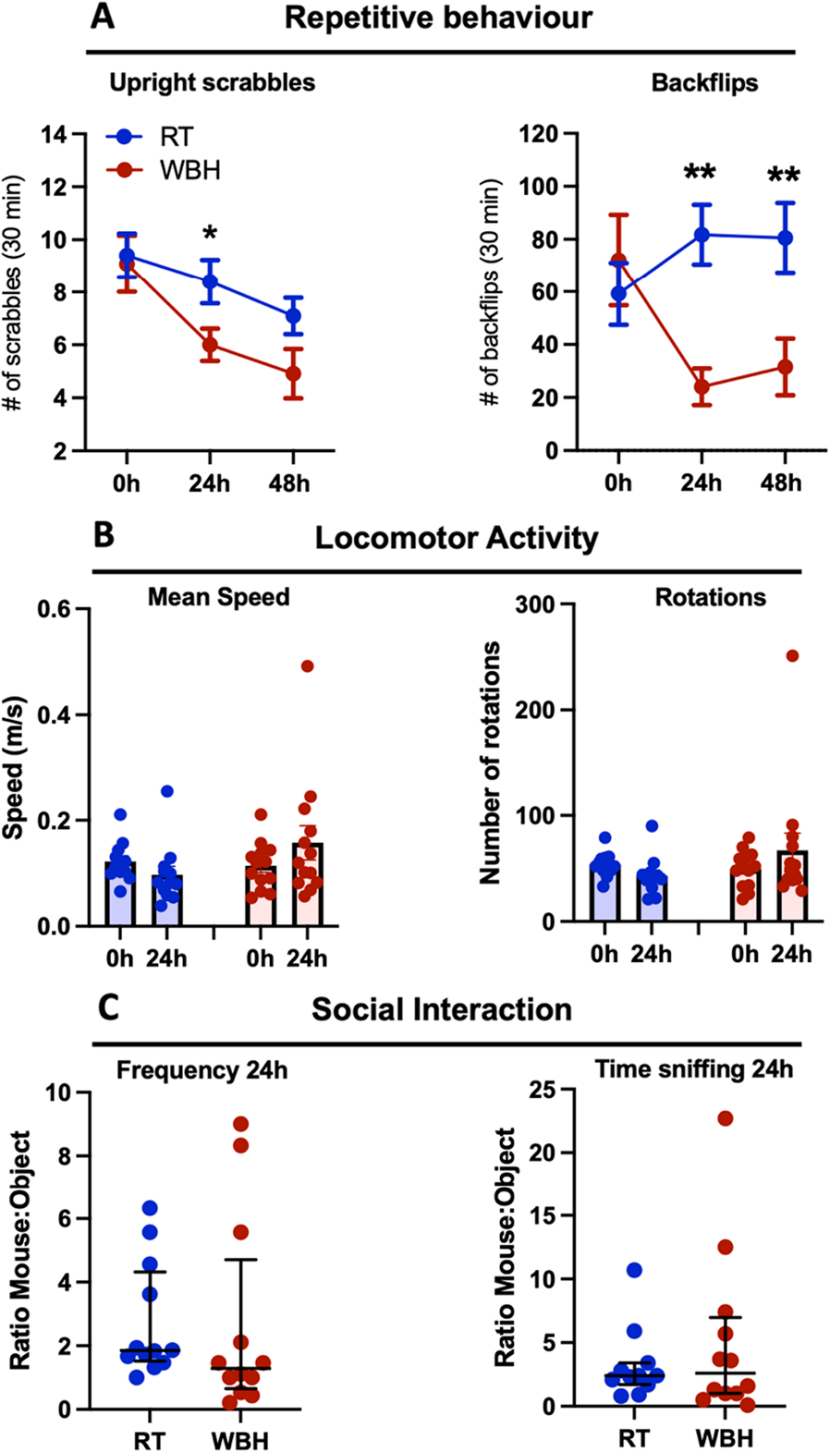
Persistent effects of WBH on behavioural deficits in the C58 mice. Timeline of the whole-body hyperthermia protocol and the behavioural tasks is as per Fig. 6F. The heating chamber was set to 33.5 °C to reach the target body temperature of 39.5 °C. **A** Effects of WBH in C58 strain on positive symptoms, over 48 h, represented by upright scrabbles and backflips. **B** Effect of WBH on locomotor activity as measured by mean speed and number of rotations. **C** Effect of WBH on social interaction as measured by frequency of sniffs and time spent sniffing an unfamiliar conspecific. All data are presented as mean ± SEM (n = 12 for WBH and 11 for RT) and * and ** denote p < 0.05 and p < 0.01, respectively, for Fisher’s LSD post hoc comparisons of WBH vs. RT after significant main effects of treatment or time by repeated measures two-way ANOVA

Some of these behavioural changes also persisted across the 24 or 48 h examined here. WBH-treated C58 mice showed significantly lower levels of the repetitive behaviours backflips and upright scrabbles indicating an improvement in these key ‘positive symptoms’. Examining data at 0, 24 and 48 h by repeated measures two-way ANOVA, we found a main effect of WBH treatment for backflips (*F*_1,20_ = 5.99; *p* = 0.0237) and this repetitive behaviour was significantly lower in WBH mice than in RT mice at both 24 h and 48 h (*p* = 0.0017 and *p* = 0.0069, respectively, by Fisher’s LSD post hoc comparison). There was a significant main effect of treatment (*F*_1,20_ = 4.75, *p* = 0.0413) and of time on upright scrabbles (*F*_2,40_ = 7.78, *p* = 0.0014) and WBH mice showed significantly less of this repetitive behaviour than RT mice at 24 h (*p* = 0.0327) but no longer at 48 h (*p* = 0.0752). Two-way ANOVA showed no significant effect of WBH on open-field speed (*F*_1,21_ = 0.6978, *p* = 0.4129) or rotations (*F*_1,21_ = 0.653, *p* = 0.4821; Fig. 7B).

Concerning ‘negative symptoms’, there were no persistent effects of WBH on social interaction in C58 mice, whether measured by frequency of sniffs (U = 0.1948) or time sniffing (*U* = 0.9640). Thus, WBH can improve both positive (repetitive) and negative (social interaction) behaviours in the C58 model, although not all behaviours were significantly mitigated and not all effects persist at 24 and 48 h (Fig. 7).

### Shank3B- characterization in the basal state.

**Fig. 8:**
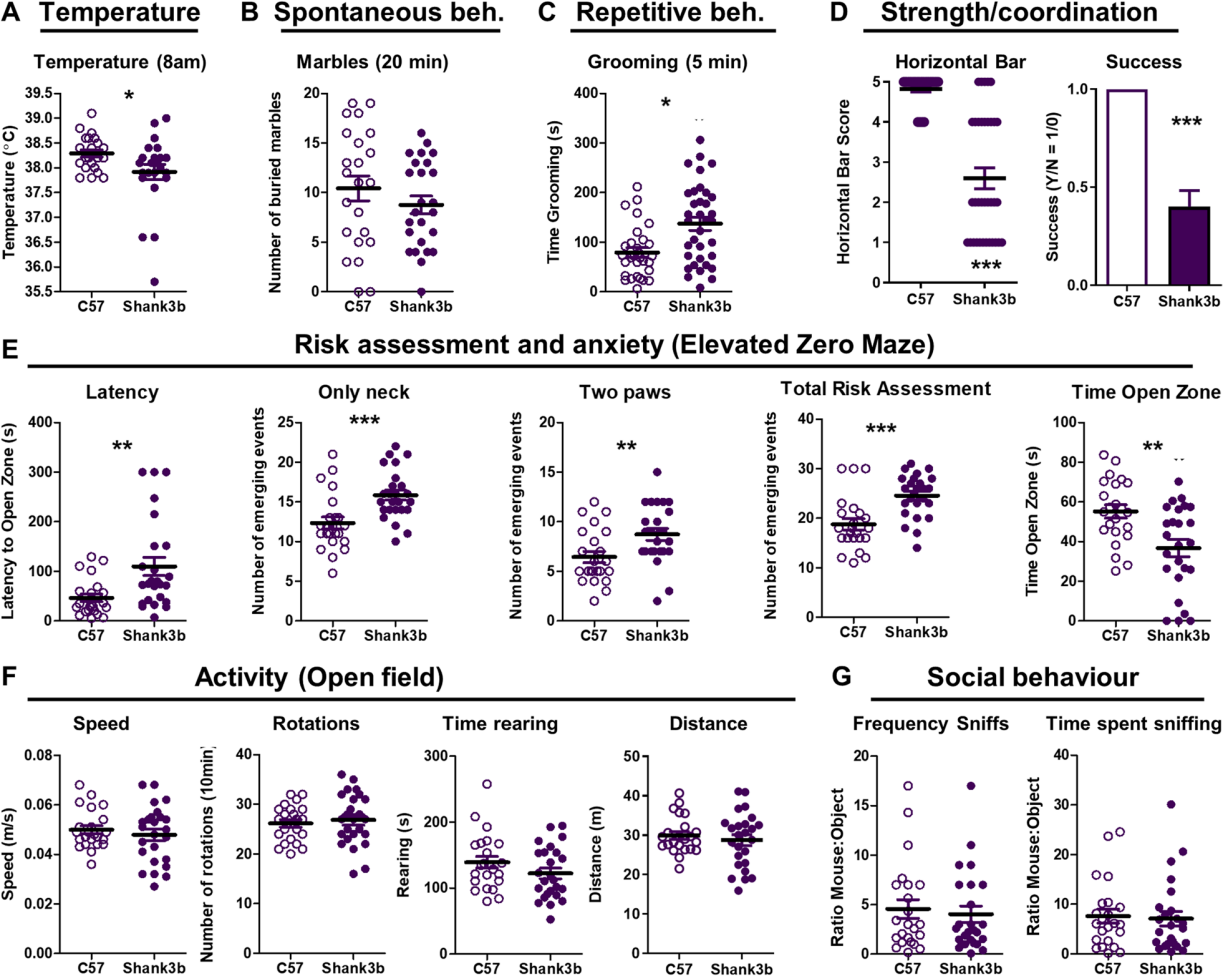
Characterization of Shank3B- mice in basal state. **A** Basal body temperature at 8am, as measured by subcutaneously implanted transponder (°C). **B** Spontaneous behaviour as measured by marbles buried in 20 min. **C** Description of the repetitive behaviours found in Shank3B- strain: grooming test for 5 min. **D** Assessment of the motor coordination and muscular strength on the horizontal bar test for 1 min (animals score 1 if they fall off within 10 s, score 2 if they hold on for 11–59 s, score 3 if they hold on for 60 s or reached the safe platform in 60 s, score 4 if they reached the safe platform within 30 s and score 5 if they reached the platform within 10 s. **E** Analyses of risk assessment and anxiety-like behaviours using the elevated zero maze (EZM) test and monitoring the latency time to go to the open zone of the maze, the number of events of risk assessment (neck, two paws and total events emerging) and the time spent in the open zone of the EZM during 5 min. **F** General and exploratory activity as assessed in the open-field test, showing the mean speed (m/s), number of rotations, time rearing (s) and travelled distance (m) for 10 min. **G** Social behaviour characterization using the frequency of sniffs and the time spent sniffing of a mouse in comparison with an inanimate object (ratio mouse:object) for 10 min. All data are expressed as mean ± SEM, *n* = 23 per group. * versus C57 strain by unpaired two-tailed t-test or Mann–Whitney, p < 0.05

In order to test whether fever-range temperature was also beneficial in another ASD-relevant strain, we analysed Shank3B- mice. We chose heterozygotes since clinical autism-associated *Shank3* mutations are heterozygous. Similar to the C58 strain characterization, a battery of strain-specific behavioural tests were run on Shank3B- mice and normal controls, at baseline, in order to assess some autism-like positive and negative symptoms previously reported for homozygous Shank3B^−/−^ mice [23–25] and to identify which of those were most robust and suitable for repeat testing in subsequent experiments. Some, but not all, of those previously reported were observed here. Body temperature (at 8am) was significantly lower in Shank3B- mice than in C57 mice (Mann–Whitney; *p* = 0.0467; Fig. 8A). There was no significant difference between the 2 strains on marble burying [34] (Fig. 8B). Shank3B- mice are known to show characteristic excessive and repetitive grooming behaviour [23–25] which we also observed here, as increased time spent grooming (Mann–Whitney; *p* = 0.0278) in Shank3B- mice (Fig. 8C).

Muscle strength and coordination were assessed using the horizontal bar test [35] and Shank3B- mice scored significantly lower in this test on a 5-point scale (see legend; Mann–Whitney; *p* < 0.0001) and also showed worse performance when categorized by success/failure to reach the safe platform (Mann–Whitney; *p* < 0.0001; Fig. 8D). The elevated zero maze (EZM) was used to assess anxiety-like behaviours and the trade-off between motivation to explore and tendency to remain in the less anxiogenic closed area [36]. At basal levels, Shank3B- mice showed higher latency to go to the open zone of the EZM (Mann–Whitney; *p* = 0.0012), spent less time in the open zone (*p* = 0.0017; Fig. 8E) and showed a higher frequency of risk assessment (see Additional file 1: Table 1 for full statistical analysis). General and exploratory activity were not significantly different between strains (Fig. 8F). We saw no significant difference in social interaction (Fig. 8G). This battery identifies several useful measures with which to assess the effect of WBH on behavioural features of the Shank3B- strain. Backflips and upright scrabbles, previously reported in the C58 strain (Figs. 6 and 7) were never observed in any Shank3B mouse in this study, whether before or after the WBH protocol and were not pursued in the WBH Shank3B experiments.

### Effects of WBH on Shank3B- strain

**Fig. 9:**
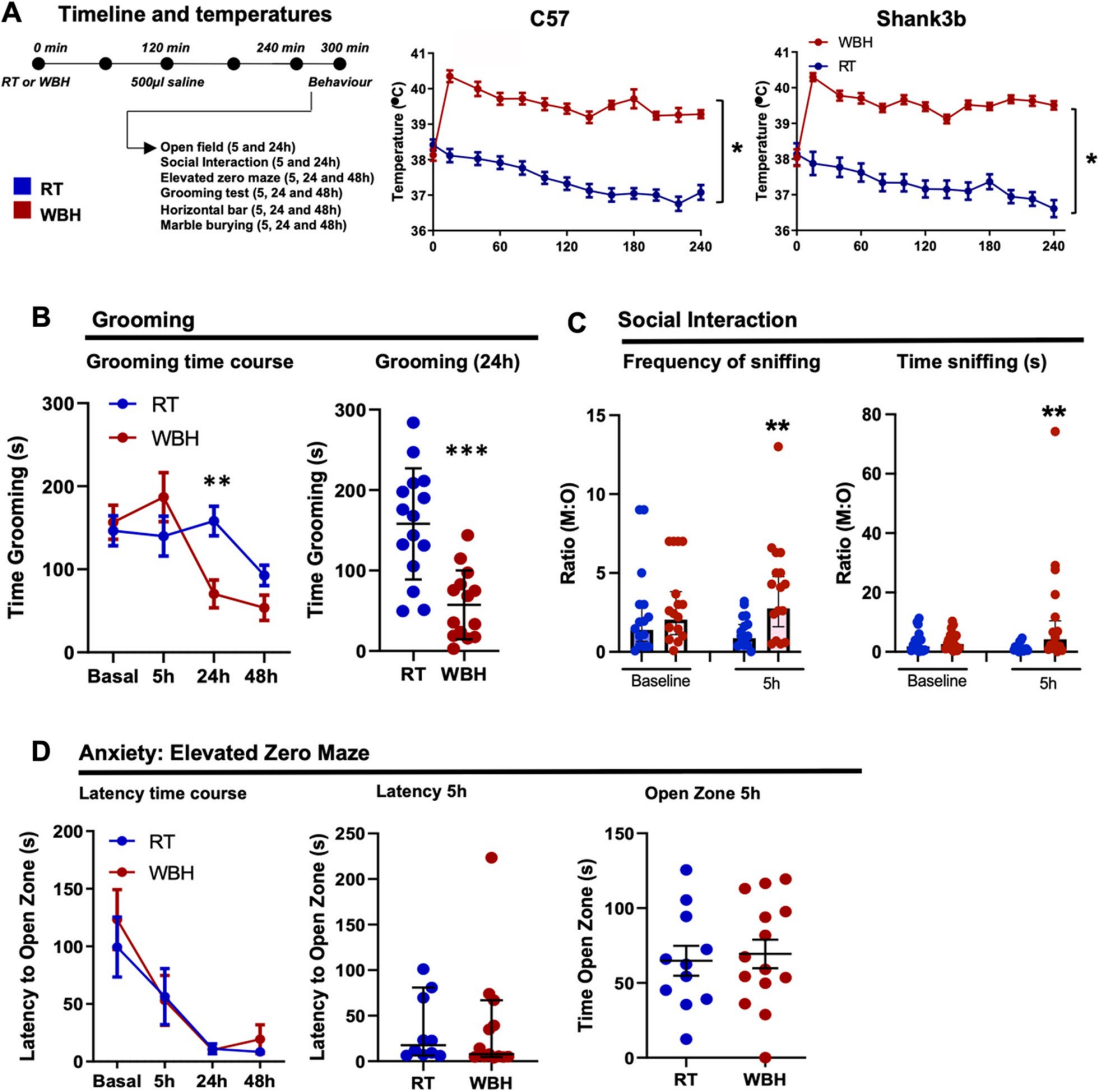
Effect of WBH on behaviour in Shank3B- mice. **A** Schematic timeline of the WBH protocol and behavioural tasks undertaken (left) and the impact of that protocol on body temperature (°C) over the five hours duration of the protocol, measured by subcutaneous temperature transponders in C57 and Shank3B- mice (right). **B** Effect of WBH on time spent grooming across the full time course and, with individual animals shown, at 24 h after heating protocol. **C** Effect of WBH (5 h) on social interaction tasks: frequency of sniffs and time spent sniffing. **D** Effect of WBH on latency to enter the open zone and time spent on the open zone assessed by the elevated zero maze in Shank3B-, shown across time and with individual animals shown at 5 h. Data are presented as mean ± SEM (**A**, **B**, **D**) or median ± IQR (**C** and **D**, centre panel) (Shank3B- RT, WBH: *n* = 15,16, respectively, except for EPM test: *n* = 11,14, respectively). Data were analysed by repeated measures two-way ANOVA (**A**, **B**, **D**) and Kruskal–Wallis (**C**). Post hoc pairwise comparisons were made using Bonferroni for parametric analyses and Dunn for nonparametric analyses. Mann–Whitney U tests were performed for simple pairwise comparison of WBH and RT at 5 or 24 h. p < 0.05, 0.01 and 0.001 for WBH vs. RT group are denoted by *, ** and ***, respectively

In independent experiments, using the standard hyperthermia protocol (i.e. chamber temperature of 38.5 ± 0.5 °C), Shank3B- animals and C57 control mice reached the target temperature of 39.5 ± 0.5 °C (Fig. 9A) and maintained this during the 4 h of the protocol (significant effect of WBH treatment in C57 and Shank3B- mice (*F*_1,27_ = 143.1 and *F*_1, 33_ = 93.27, respectively; *p* < 0.0001).

Repetitive and excessive grooming is one of the characteristic alterations in Shank3B- mice, sometimes leading to skin lesions and scarring, although this severity was not observed here. Indeed, a small number of individuals showed grooming clearly below average C57 control levels and were not analysed for WBH effects. As was observed with C57 and C58 mice, all mice showed elevated grooming immediately upon emergence from the heating protocol, but this resolved rapidly (Additional file 1: data 4). Therefore, analysis of grooming was focussed on 24 and 48 h. The excessive grooming visible at baseline was significantly mitigated after WBH (Fig. 9B). In repeated measures two-way ANOVA, there was a main effect of treatment (*F*_1,28_ = 5.46, *p* = 0.0268) and an interaction of treatment and time (*F*_2,56_ = 5.25, *p* = 0.0081). Bonferroni post hoc test showed a significant difference between WBH and RT at 24 h (*p* = 0.0014) but no longer at 48 h.

Although social interaction did not show significant differences between Shank3B- and C57BL6 mice at baseline (Fig. 8G), given its high relevance to ASD, we did measure this following WBH. Shank3B mice were pseudorandomized to treatment (RT or WBH) such that they were balanced for baseline performance. We found that RT Shank3B- mice performed worse in consecutive social interaction tests, whereas WBH group showed no such worsening or even a slight improvement after the heating protocol. Therefore, using Kruskal–Wallis analysis for these nonparametric data, we found significant effects of treatment for both frequency of sniffing (*p* = 0.0336) and time sniffing (*p* = 0.0141) and significant pairwise comparison at 5 h post-WBH for both frequency (*p* = 0.0062) and time sniffing (*p* = 0.0054). These data warrant caution since this difference does not constitute an improvement from baseline, but does indicate better social interaction 5 h after WBH than in those 5 h after exposure to the same arena without heating.

Although Shank3B- showed higher latency to exit the closed area in EZM (Fig. 8E), this task was ill-suited to retesting over 48 h since the maze became less anxiogenic on repeated exposures in all mice (Fig. 9D). Thus, it was not feasible to demonstrate an effect of WBH on this measure of anxiety. Open-field activity, marble burying and muscle strength/coordination on the horizontal bar were also assessed here, but none showed significant changes arising from the WBH protocol (Additional file 1: Fig. 5).

## Discussion

In the present study, we have experimentally dissociated the inflammatory and hyperthermic components of fever and show, for the first time, that raising body temperature to 39.5 ± 0.5 °C for 4 h, in the absence of an inflammatory stimulus, improves behavioural signs in two distinct mouse models of ASD. Although hyperthermia is not a viable therapeutic strategy for treating individuals with ASD, and is not recommended by the current results, these findings should stimulate mechanistic studies to understand molecular pathways through which hyperthermia may produce the observed improvements.

### WBH alleviates strain-dependent features in C58/J and Shank3B- mice

WBH mitigated some behavioural and physiological features in the widely used C58/J and Shank3B- models of ASD, although not improving all behavioural features, and not completely reversing those that were improved. Given that ASD comprises conditions that are largely genetically determined and developmental in nature, any environmental change that can improve function deserves interrogation.

In the two strains examined here, the WBH protocol significantly decreased the most consistent repetitive behaviours associated with both of those specific models. That is, backflipping and upright scrabbles in C58/J mice and excessive grooming in Shank3B- mice. Both backflipping in C58/J mice and excessive grooming were reduced by more than 50% and significant effects persisted on grooming and upright scrabbles at 24 h and on backflips at 48 h, long after animals had returned to normal body temperature. Given that fever-associated improvements were observed in ASD individuals (i.e. humans) with more severe repetitive behaviours [2], the current findings may be significant. However, the reduction of hyperactive (or positive) behaviours is not, of itself, sufficient to demonstrate improvement in ASD-related phenotypes. This is because in the initial fever studies [1] individuals with fever typically also experienced lethargy and this might have contributed to apparent improvements in repetitive behaviours. Our studies with LPS in C57 mice (Fig. 5) clearly show suppression of spontaneous behaviour during acute inflammation, making this stimulus unsuitable for assessment of ‘improvements’ in ASD-related hyperactive/repetitive behaviours (‘positive symptoms’). Given the scope for non-specific suppression of general activity, it is significant that WBH could also trigger improvements in ‘negative symptoms’, i.e. behaviours that are suppressed in the ASD-related strain: we observed a significant improvement in social interaction after WBH in C58/J mice.

Notably, body temperature was lower in C58/J than C57 mice at baseline and, although the difference was more modest, Shank3B body temperature was also lower than C57 at baseline. This poses a question as to whether baseline temperature differences might influence strain-dependent differences in behaviour. Using a novel thermocouple, we found that the brain temperature increased temporally in synchronization with, and proportionately to, elevated body temperature. In general, brain temperature was approximately 1.5ºC higher than body temperature independently of the treatment (RT, LPS or WBH), but rose in tandem with hyperthermia. Brain temperature has been shown to be approximately 1.5 °C higher than arterial blood, to fluctuate across the circadian clock and to change with environmental stimulation [37]. Increased brain temperature in the dark phase, and upon initiation of activity, influences hippocampal neurophysiological activity [38], raising the temperature of hippocampal slices increases excitatory postsynaptic potentials [39] and AMPA receptors show more rapid postsynaptic AMPA receptor kinetics [40]. How these changes affect synaptic summation in a complex network is more difficult to model and predictions for behaviour are unclear. Nonetheless, in the current study, raising body and brain temperature by approximately 1.5–2 °C improved several behavioural indices in two ASD models and interrogating whether this elevation of brain temperature is necessary for the behavioural effects observed now requires investigation.

### Dissociation of hyperthermia from inflammation

Although some ASD features have been suggested to improve during fever in children, inflammation produces sickness behavioural changes including the general suppression of spontaneous activity [29], which might account for apparent improvements in ‘positive symptoms’ (i.e. behaviours that show higher frequency in ASD cases). Here, we deliberately dissociated hyperthermia from inflammation and confirmed that while LPS-treated animals showed the expected general suppression of spontaneous activity but did not mitigate either positive or negative symptoms, there were benefits of elevated temperature alone. More recent studies suggests that very few children benefit from febrile episodes [2, 41], but the examination of individual features revealed effects consistent with sickness behaviour responses (increased sleep, negative effects on activity, feeding and happiness). Thus, acute illness per se is unlikely to provide improvements for significant numbers of individuals with ASD. However, whether elevated body temperature alone may provide benefits is a distinct question. The WBH protocol, adapted from previously published WBH experiments [10, 42] increased body temperature and maintained it at 39.5 ± 0.5 °C for the full 4 h hours of heating without inducing inflammation. Conversely, only LPS (250 µg/Kg) induced pro-inflammatory cytokines in peripheral blood and in the brain (Fig. 2), but temperature in LPS animals never reached the fever range. Thus, hyperthermia and inflammation were fully dissociable in our experimental manipulations.

It has recently been shown that LPS-induced inflammation improved social behaviour in mice arising from an in utero ‘maternal immune activation’ (MIA) model of ASD [43]. Those improvements did not occur in monogenic models, including Shank3B-. The behavioural improvements were dependent on the cytokine IL-17 and it is possible that ASD cases arising from MIA may selectively show beneficial effects of infection mimetics since responses to acute inflammatory stimuli, including induction of IL-17a, are influenced by prior inflammatory exposures in utero [44, 45]*.* The authors excluded an effect of fever per se in their LPS/MIA studies since elevation of body temperature by LPS or using a DREADD approach (designer receptors exclusively activated by designer drugs), did not produce similar behavioural improvements. However, the increases in body temperature in the Reed study were modest: the authors state that LPS ‘led to a significant increase in body temperature (of about 0.5–1.0 °C)’, but temperatures always remained below 37 °C. It may be important that baseline temperatures were also approximately 1.0 °C lower in those mice than in our studies, which may result from the different measurement approach and/or vivarium conditions, but with temperatures in the DREADD experiments also reaching only 36.5 °C, we contend that those data do not rule out that febrile responses may improve behaviour in one or more ASD models. Our studies are distinct from those of Reed et al. in important ways. 1) We observed improvements in behaviour in both C58/J and Shank3B- models after animals reached typical fever-range body temperatures of 39–40 °C, 2) The LPS dose we used was significantly higher than that used by Reed et al., (250 µg/Kg vs. 50 µg/Kg). It was our explicit aim to induce inflammation sufficient to produce lethargy/hypoactivity (Fig. 5) since this also occurs in the human ASD studies [2]). Reed et al., produced a more modest suppression of activity, which probably facilitated maintenance of social activity. Our findings with LPS are consistent the observation that acute illness per se provides limited benefit for most ASD patients [41], but we suggest that elevated temperature may trigger molecular changes that do offer benefits, independent of inflammation, and these now require investigation.

### Strain considerations and potential convergence on synaptic function

Most studies using Shank3B- mutants have compared null mutants to wildtype littermate controls [24–26, 46]. However, ASD cases, particularly those with Phelan–McDermid syndrome [47] are largely haploinsufficient rather than null [48]. This is sufficient to convey a high risk for ASD in humans [49] and for synaptic deficits, altered social interaction and repetitive behaviours [50–52] in mice. Here, using heterozygotes, Shank3B- phenotypes were largely still evident, although we did not observe clear social impairments. The most robust deficit we did observe, excessive grooming, was significantly mitigated by fever-range temperature. The basis of this temperature-induced improvement has not been determined here.

It is known that Shank3B is a scaffolding protein that facilitates interaction with NMDA, AMPA and metabotropic glutamate receptors in glutamatergic dendrites. Shank3B mutants have shown altered dendritic structure and enhanced inhibitory tone [46, 50]. The genetic basis of neuronal circuit dysfunction in the inbred strain, C58/J, is unclear, but there are also changes in dendritic structure and both repetitive and social interaction deficits in C58/J mice were mitigated by mGluR5 negative allosteric modulation [53]. It may be of interest to investigate whether increasing temperature may modulate dendritic structure and/or glutamatergic transmission in these two strains.

### Pathways of cellular and molecular activation

Although we have not described the cellular or molecular mechanisms for temperature-driven mitigations of behavioural deficits in the C58 and Shank3B- mice, cFos labelling does point to other possibilities for follow-up studies. Regions previously described in a ‘warm sensitive circuit’ that responds to elevations in internal or external temperature [14] were clearly activated: anterior and preoptic areas of the hypothalamus mediate thermoregulatory homeostasis and lateral septum, medial habenula (mHab), BNST and PVN also participate in this circuitry. In addition to the PVN, VMPO and LHab, which were activated 4 h after WBH and LPS, cFOS labelling identified other nuclei selectively activated by WBH (LSV, DMH), while the CEA was only significantly activated by LPS. Some of these regions form part of the extended amygdala circuit, involved in anxiety, fear, salience and reward [54, 55]. The PVN, amygdala and hippocampus are implicated in emotional and memory contributions to social interaction [56–58] and the habenula plays an important role in social interaction [59].

There is also evidence that warm temperature can alleviate some symptoms in those with major depression [60–62]. Although the mechanisms are entirely unclear, heating led to activation of LS, NAc, mHab, dorsal hypothalamus and other regions [62]. It will be of interest to use optogenetic or DREADD approaches to experimentally activate selected brain regions identified herein with a view to simulating the positive effects of fever on ASD-related behavioural abnormalities.

Hypoglycaemia developed by 4 h under both LPS and WBH treatments (Fig. 4A, B) and this is shown to trigger reduced spontaneous activity [29]. Acute hypoglycaemia drives elevated blood ketones and ketone diets have been suggested to be beneficial in some ASD cases [63]. Given the multiple bioenergetic changes that may occur during elevated ketone levels, it may be informative to assess ketogenesis during WBH and to assess general impacts on brain bioenergetic function.

### Limitations and conclusions

The current study had some significant limitations. Firstly, it would be beneficial to continue behavioural assessment well beyond 48 h to assess whether there may be some long-term benefits or even deleterious consequences of the treatment. More importantly, we provide only a limited analysis of the cellular and molecular changes during hyperthermia and do not examine what molecular mechanisms explain the behavioural improvements. However, there are also significant strengths. The positive effects of the WBH protocol were observed in two different ASD-relevant mouse strains that are each characterized by different behavioural phenotypes at baseline. The findings are strengthened by the fact that the WBH protocol, which produced hyperthermia for 4 h without an inflammatory stimulus, was sufficient to improve both positive and negative, strain-specific, behavioural phenotypes. The fact that the observed improvements are not confounded by a generalized suppression of spontaneous activity, which occurs during acute LPS-induced systemic inflammation, a form of which would usually accompany the fever response, gives confidence that it is the elevated brain/body temperature per se that supports the improvements in behaviour.

Integrating our findings with those of the Reed study [43] and with previous human studies [1, 2], we propose that the presence or absence of improvements under hyperthermic conditions will likely depend on the initial aetiology of the ASD and on the severity of inflammation and fever occurring. However, given the potential of pyrogenic pro-inflammatory mediators such as IL-1β, IL-6 and prostaglandins to suppress motivation, mood, social activity, arousal and cognition [7], it remains plausible that the effects of elevated temperature could actually be more impressive if elevated temperature should occur in the absence of inflammation. It would be beneficial, in human ASD cases experiencing infections, to measure body temperature and metabolic parameters in order to come to more robust conclusions about the relationships between fever, inflammation and ASD symptoms.

### Supplementary Information

**Additional file 1**. Supplementary tables and figures.

## Data Availability

Data will be made available upon reasonable request to the authors.

## Abbreviations

AMPA: α-Amino-3-hydroxy-5-methyl-4-isoxazolepropionic acid
ANKRD11: Ankyrin repeat domain 11
ANOVA: Analysis of variance
ASD: Autism spectrum disorder
AUC: Area under the curve
BLA: Basolateral amygdala
BNST: Bed nucleus of the stria terminalis
cDNA: Complementary deoxyribonucleic acid
Cox2: Cyclooxygenase 2
CT: Cycle threshold
DMH: Dorsomedial hypothalamic nucleus
DREADD: Designer receptors exclusively activated by designer drugs
DRI: Interfascicular dorsal raphe
ELISA: Enzyme-linked immunoassay
EZM: Elevated zero maze
GRM7: Metabotropic glutamate receptor 7
i.p.: Intraperitoneal
Igf1: Insulin-like growth factor 1
IL: Interleukin
Il1b: Interleukin 1 beta
Il6: Interleukin 6
LPS: Lipopolysaccharide
LS: Lateral septum
LTP: Long-term potentiation
MAP1A: Microtubule-associated protein 1A
mGluR5: Metabotropic glutamate receptor 5
mHab: Medial habenula
MIA: Maternal immune activation
MS: Medial septum
NMDA: N-methyl-D-aspartate
OD: Optical density
PBS: Phosphate-buffered saline
PCR: Polymerase chain reaction
PFA: Paraformaldehyde
PSD95: Postsynaptic density—95 KDa
PTZ: Pentylenetetrazole
PVN: Paraventricular nucleus
Rankl: Receptor activator of nuclear factor kappa beta ligand
RNA: Ribonucleic acid
RT: Room temperature
RT-PCR: Real-time PCR
SEM: Standard error of the mean
TNF-α: Tumour necrosis factor alpha
VMPO: Ventromedial preoptic nucleus
WBH: Whole-body hyperthermia

## References

[1] Curran LK, Newschaffer CJ, Lee L-CC, Crawford SO, Johnston MV, Zimmerman AW (2007). Behaviours associated with fever in children with autism spectrum disorders. Pediatrics.

[2] Grzadzinski R, Lord C, Sanders SJ, Werling D, Bal VH (2018). Children with autism spectrum disorder who improve with fever: insights from the Simons Simplex Collection. Autism Res..

[3] Van Hook MJ (2020). Temperature effects on synaptic transmission and neuronal function in the visual thalamus. PLoS One.

[4] Long MA, Fee MS (2008). Using temperature to analyse temporal dynamics in the songbird motor pathway. Nature.

[5] Mrozek S, Vardon F, Geeraerts T (2012). Brain temperature: physiology and pathophysiology after brain injury. Anesthesiol Res Pract.

[6] Nunneley SA, Martin CC, Slauson JW, Hearon CM, Nickerson LDH, Mason PA (1985). Changes in regional cerebral metabolism during systemic hyperthermia in humans. J Appl Physiol.

[7] Saper CB, Romanovsky AA, Scammell TE (2012). Neural circuitry engaged by prostaglandins during the sickness syndrome. Nat Neurosci.

[8] Rudaya AY, Steiner AA, Robbins JR, Dragic AS, Romanovsky AA (2005). Thermoregulatory responses to lipopolysaccharide in the mouse: dependence on the dose and ambient temperature. Am J Physiol Regul Integr Comp Physiol.

[9] Skelly DT, Hennessy E, Dansereau M-A, Cunningham C (2013). A systematic analysis of the peripheral and CNS effects of systemic LPS, IL-1β, [corrected] TNF-α and IL-6 challenges in C57BL/6 mice. PLoS One.

[10] Evans SS, Repasky EA, Fisher DT (2015). Fever and the thermal regulation of immunity: the immune system feels the heat. Nat Rev Immunol.

[11] Wilkinson DA, Burholt DR, Shrivastava PN (1988). Hypothermia following whole-body heating of mice: effect of heating time and temperature. Int J Hyperth.

[12] Cunningham C, Wilcockson DC, Campion S, Lunnon K, Perry VH (2005). Central and systemic endotoxin challenges exacerbate the local inflammatory response and increase neuronal death during chronic neurodegeneration. J Neurosci.

[13] Dallaporta M, Pecchi E, Jacques C, Berenbaum F, Jean A, Thirion S (2007). c-Fos immunoreactivity induced by intraperitoneal LPS administration is reduced in the brain of mice lacking the microsomal prostaglandin E synthase-1 (mPGES-1). Brain Behav Immun.

[14] Tan CL, Cooke EK, Leib DE, Lin YC, Daly GE, Zimmerman CA (2016). Warm-sensitive neurons that control body temperature. Cell.

[15] Lacroix S, Rivest S (1997). Functional circuitry in the brain of immune-challenged rats: partial involvement of prostaglandins - PubMed. J Comp Neurol.

[16] Deacon RMJ (2006). Digging and marble burying in mice: simple methods for in vivo identification of biological impacts. Nat Protoc.

[17] Deacon RMJ (2009). ` sensitive behavioural assay, tested in five species of laboratory rodents. Behav Brain Res.

[18] Blick MG, Puchalski BH, Bolanos VJ, Wolfe KM, Green MC, Ryan BC (2015). Novel object exploration in the C58/J mouse model of autistic-like behaviour. Behav Brain Res.

[19] Moy SS, Nadler JJ, Young NB, Nonneman RJ, Segall SK, Andrade GM (2008). Social approach and repetitive behaviour in eleven inbred mouse strains. Behav Brain Res.

[20] Moy SS, Riddick NV, Nikolova VD, Teng BL, Agster KL, Nonneman RJ (2014). Repetitive behaviour profile and supersensitivity to amphetamine in the C58/J mouse model of autism. Behav Brain Res.

[21] Muehlmann AM, Edington G, Mihalik AC, Buchwald Z, Koppuzha D, Korah M (2012). Further characterization of repetitive behaviour in C58 mice: developmental trajectory and effects of environmental enrichment. Behav Brain Res.

[22] Whitehouse CM, Curry-Pochy LS, Shafer R, Rudy J, Lewis MH (2017). Reversal learning in C58 mice: modeling higher order repetitive behaviour. Behav Brain Res.

[23] Balaan C, Corley MJ, Eulalio T, Leite-ahyo K, Pang APS, Fang R (2019). Juvenile Shank3b deficient mice present with behavioural phenotype relevant to autism spectrum disorder. Behav Brain Res.

[24] Peça J, Feliciano C, Ting JT, Wang W, Wells MF, Venkatraman TN (2011). Shank3 mutant mice display autistic-like behaviours and striatal dysfunction. Nature.

[25] Mei Y, Monteiro P, Zhou Y, Kim JA, Gao X, Fu Z (2016). Adult restoration of Shank3 expression rescues selective autistic-like phenotypes. Nature.

[26] Wang X, McCoy PA, Rodriguiz RM, Pan Y, Je HS, Roberts AC (2011). Synaptic dysfunction and abnormal behaviours in mice lacking major isoforms of Shank3. Hum Mol Genet.

[27] Murray C, Sanderson DJ, Barkus C, Deacon RMJ, Rawlins JNP, Bannerman DM (2012). Systemic inflammation induces acute working memory deficits in the primed brain: relevance for delirium. Neurobiol Aging.

[28] Thorne AM, Ubbink R, Bruggenwirth IMA, Nijsten MW, Porte RJ, de Meijer VE (2020). Hyperthermia-induced changes in liver physiology and metabolism: a rationale for hyperthermic machine perfusion. Am J Physiol Gastrointest Liver Physiol.

[29] Kealy J, Murray C, Griffin EW, Lopez-Rodriguez AB, Healy D, Tortorelli LS (2020). Acute inflammation alters brain energy metabolism in mice and humans: role in suppressed spontaneous activity, impaired cognition, and delirium. J Neurosci.

[30] Vahdatpour C, Dyer AH, Tropea D (2016). Insulin-like growth factor 1 and related compounds in the treatment of childhood-onset neurodevelopmental disorders. Front Neurosci.

[31] Cataldo I, Azhari A, Esposito G (2018). A review of oxytocin and arginine-vasopressin receptors and their modulation of autism spectrum disorder. Front Mol Neurosci.

[32] Ryan BC, Young NB, Crawley JN, Bodfish JW, Moy SS (2010). Social deficits, stereotypy and early emergence of repetitive behaviour in the C58/J inbred mouse strain. Behav Brain Res.

[33] Jirkof P (2014). Burrowing and nest building behaviour as indicators of well-being in mice. J Neurosci Methods.

[34] Thomas A, Burant A, Bui N, Graham D, Yuva-Paylor LA, Paylor R (2009). Marble burying reflects a repetitive and perseverative behaviour more than novelty-induced anxiety. Psychopharmacology.

[35] Deacon RMJ (2013). Measuring motor coordination in mice. J Vis Exp.

[36] Deacon RMJ (2013). The successive alleys test of anxiety in mice and rats. JoVE (J Vis Exp).

[37] Kiyatkin EA, Brown PL, Wise RA (2002). Brain temperature fluctuation: a reflection of functional neural activation. Eur J Neurosci.

[38] Petersen PC, Voroslakos M, Buzsáki G (2022). Brain temperature affects quantitative features of hippocampal sharp wave ripples. J Neurophysiol.

[39] Schiff SJ, Somjen GG (1985). The effects of temperature on synaptic transmission in hippocampal tissue slices. Brain Res.

[40] Postlethwaite M, Hennig MH, Steinert JR, Graham BP, Forsythe ID (2007). Acceleration of AMPA receptor kinetics underlies temperature-dependent changes in synaptic strength at the rat calyx of Held. J Physiol.

[41] Byrne K, Zheng S, Bishop S, Boucher J, Ghods S, Kim SH, et al. Behavioural responses to fevers and other medical events in children with and without ASD. medRxiv. 2022 ;2022.05.23.22275374. 10.1002/aur.2810 36164255

[42] Ostberg JR, Taylor SL, Baumann H, Repasky EA (2000). Regulatory effects of fever-range whole-body hyperthermia on the LPS-induced acute inflammatory response. J Leukoc Biol.

[43] Reed MD, Yim YS, Wimmer RD, Kim H, Ryu C, Welch GM (2020). IL-17a promotes sociability in mouse models for neurodevelopmental disorders. Nature.

[44] Meyer U (2014). Prenatal poly(i:C) exposure and other developmental immune activation models in rodent systems. Biol Psychiatry.

[45] Neher JJ, Cunningham C (2019). Priming microglia for innate immune memory in the brain. Trends Immunol.

[46] Dhamne SC, Silverman JL, Super CE, Lammers SHT, Hameed MQ, Modi ME (2017). Replicable in vivo physiological and behavioural phenotypes of the Shank3B null mutant mouse model of autism. Mol Autism.

[47] Phelan K, McDermid HE (2012). The 22q13.3 deletion syndrome (Phelan-McDermid Syndrome). Mol Syndromol.

[48] Betancur C, Buxbaum JD (2013). SHANK3 haploinsufficiency: a “common” but underdiagnosed highly penetrant monogenic cause of autism spectrum disorders. Mol Autism.

[49] Leblond CS, Nava C, Polge A, Gauthier J, Huguet G, Lumbroso S (2014). Meta-analysis of SHANK mutations in autism spectrum disorders: a gradient of severity in cognitive impairments. PLoS Genet.

[50] Bozdagi O, Sakurai T, Papapetrou D, Wang X, Dickstein DL, Takahashi N (2010). Haploinsufficiency of the autism-associated Shank3 gene leads to deficits in synaptic function, social interaction, and social communication. Mol Autism.

[51] Duffney LJ, Zhong P, Wei J, Matas E, Cheng J, Qin L (2015). Autism-like deficits in Shank3-deficient mice are rescued by targeting actin regulators. Cell Rep.

[52] Qin L, Ma K, Wang ZJ, Hu Z, Matas E, Wei J (2018). Social deficits in Shank3-deficient mouse models of autism are rescued by histone deacetylase (HDAC) inhibition. Nat Neurosci.

[53] Silverman JL, Smith DG, Rizzo SJS, Karras MN, Turner SM, Tolu SS (2012). Negative allosteric modulation of the mGluR5 receptor reduces repetitive behaviours and rescues social deficits in mouse models of Autism. Sci Transl Med.

[54] Shackman AJ, Fox AS (2016). Contributions of the central extended amygdala to fear and anxiety. J Neurosci.

[55] Stamatakis AM, Sparta DR, Jennings JH, Mcelligott ZA, Decot H, Stuber GD (2014). Amygdala and bed nucleus of the stria terminalis circuitry: Implications for addiction-related behaviours. Neuropharmacology.

[56] Young WS, Li J, Wersinger SR, Palkovits M (2006). The vasopressin 1b receptor is prominent in the hippocampal area CA2 where it is unaffected by restraint stress or adrenalectomy. Neuroscience.

[57] Wang Y, Zhao S, Liu X, Fu Q (2014). Effects of the medial or basolateral amygdala upon social anxiety and social recognition in mice. Turk J Med Sci.

[58] Tzakis N, Holahan MR (2019). Social memory and the role of the hippocampal CA2 region. Front Behav Neurosci.

[59] van Kerkhof LWM, Damsteegt R, Trezza V, Voorn P, Vanderschuren LJMJ (2013). Functional integrity of the habenula is necessary for social play behaviour in rats. Eur J Neurosci.

[60] Hanusch KU, Janssen CH, Billheimer D, Jenkins I, Spurgeon E, Lowry CA (2013). Whole-body hyperthermia for the treatment of major depression: associations with thermoregulatory cooling. Am J Psychiatry.

[61] Janssen CW, Lowry CA, Mehl MR, Allen JJB, Kelly KL, Gartner DE (2016). Whole-body hyperthermia for the treatment of major depressive disorder: a randomized clinical trial. JAMA Psychiat.

[62] Hale MW, Raison CL, Lowry CA (2013). Integrative physiology of depression and antidepressant drug action: implications for serotonergic mechanisms of action and novel therapeutic strategies for treatment of depression. Pharmacol Ther.

[63] Li Q, Liang J, Fu N, Han Y, Qin J (2021). A Ketogenic diet and the treatment of autism spectrum disorder. Front Pediatr.

