# Supplementary material for: Hyperthermia elevates brain temperature and improves behavioural signs in animal models of autism spectrum disorder
Source: Mol Autism. 2023 Nov 15;14:43. doi: 10.1186/s13229-023-00569-y (PMC10652497; doi:10.1186/s13229-023-00569-y)
Supplement: Supplementary file 1 — Additional file 1. Supplementary tables and figures. [file 13229_2023_569_MOESM1_ESM.docx]

**Additional file**

This additional file contains 2 tables and 5 figures of data that support the main figures of the paper but are not essential within the main text. Each figure below has its own figure legend and accompanying results text.

**Table S1** Full description of experimental design and statistical analyses for all figures in the main manuscript.

| **Figure** | **Group (n)** | **Test used** | **Statistical values** | ***p* value** | **Post-hoc tests** |
| --- | --- | --- | --- | --- | --- |
| 1B | C57 mice  Body temperature  RT (24)  LPS (24)  WBH (24) | 3 x 13  Repeated measures two-way ANOVA | *Time_(0 to 240minutes)_*  *F_(12,828)_ = 40.61*  *Treatment_(RT; LPS; WBH)_*  *F_(2,69)_ = 78.60*  *Treatment x Time*  *F_(24,828)_ = 13.19* | *<0.0001*  *<0,0001*  *<0,0001* | Bonferroni  RT vs LPS p <0,0001  RT vs WBH p <0,0001  LPS vs WBH p <0,0001 |
| 1C | C57 mice RT  Real time temp.  Brain (6)  Body (6) | 2 x 15  Repeated measures two-way ANOVA | *Time_(-60 to 300minutes)_*  *F_(14,84)_ = 3.052*  *Region_(Body, Brain)_*  *F_(1,5)_ = 43.12*  *Treatment x Time*  *F_(14,84)_ = 0.6054* | p=0.0871  p=0.0012  p=0.5368 |  |
| 1C | C57 mice LPS  Real time temp.  Brain (5)  Body (5) | 2 x 15  Repeated measures two-way ANOVA | *Time_(-60 to 300minutes)_*  *F_(14,70)_ = 5.537*  *Region_(Body, Brain)_*  *F_(1,4)_ = 197.1*  *Treatment x Time*  *F_(14,70)_ = 0.6781* | p=0.0365  p=0.0001  p=0.5559 |  |
| 1C | C57 mice WBH  Real time temp.  Brain (6)  Body (6) | 2 x 15  Repeated measures two-way ANOVA | *Time_(-60 to 300minutes)_*  *F_(14,84)_ = 8.306*  *Region_(Body, Brain)_*  *F_(1,5)_ = 65.27*  *Treatment x Time*  *F_(14,84)_ = 0.7708* | p=0.0015  p=0.0005  p=0.5111 |  |
| 1C | C57 mice  Brain (17)  Body (17) | Linear regression | Goodness of fit:  r^2^ = 0.6742  y= 0,7137*x + 12,42 | p=0.0001 |  |
| 2A | C57 mice  Peripheral cytokines 4h  RT (4)  LPS (4)  WBH (5) | One-way ANOVA or Kruskal Wallis | Treatment _(RT, LPS, WBH)_  *IL-1β F_(2,12)_* = 11.508  *IL-6 F_(2,12)_* = 9.750  *TNF-a F_(2,12)_* = 16.187 | p=0.0003  p=0.004  p=0.001 | Bonferroni or Mann Whitney  LPS vs RT = 0.029  LPS vs WBH = 0.016  LPS vs RT = 0.011  LPS vs WBH = 0.008  LPS vs RT = 0.001  LPS vs WBH = 0.013 |
| 2B | C57 mice  Brain cytokines 4h  Hypothalamus  RT (4)  LPS (4)  WBH (5) | One-way ANOVA | Treatment _(RT, LPS, WBH)_  *Il1b F_(2,10)_* = 11.07  *Il6 F_(2,10)_* = 15.63  *Cox2 F_(2,10)_* = 129.5  *Rankl* *F_(2,10)_* = 15.66 | 0.0029  0.0008  <0.0001  0.0008 | Bonferroni post-hoc  LPS vs RT = 0.0069  LPS vs WBH = 0.0058  LPS vs RT = 0.0024  LPS vs WBH = 0.0016  LPS vs RT <0.0001  LPS vs WBH <0.0001  LPS vs RT = 0.0019  LPS vs WBH = 0.0019 |
| 2B | C57 mice  Brain cytokines 4h  Hippocampus  RT (4)  LPS (4)  WBH (5) | One-way ANOVA | Treatment _(RT, LPS, WBH)_  *Il1b F_(2,10)_* = 18.63  *Il6 F_(2,9)_* = 40.74  *Cox2 F_(2,10)_* = 0.2610  *Rankl* *F_(2,10)_* =33.70 | p=0.0004  <0.0001  p=0.7754  <0.0001 | Bonferroni post-hoc  LPS vs RT = 0.0029  LPS vs WBH = 0.0005  LPS vs RT <0.0001  LPS vs WBH <0.0001  LPS vs RT p=0.0001  LPS vs WBH <0.0001 |
| 2B | C57 mice  Brain cytokines 4h  Amygdala  RT (4)  LPS (4)  WBH (5) | One-way ANOVA | Treatment _(RT, LPS, WBH)_  *Il1b F_(2,9)_* = 3.922  *Il6 F_(2,10)_* = 5.080  *Cox2 F_(2,10)_* =2.278  *Rankl* *F_(2,10)_* =7.169 | p=0.0596  p=0.0300  p=0.1530  p=0.0117 | Bonferroni post-hoc  LPS vs RT p=0.0790  LPS vs WBH p=0.1401  LPS vs RT p=0.0687  LPS vs WBH p=0.0496  LPS vs RT p=0.0284  LPS vs WBH p=0.0201 |
| 3 | C57 mice  cFOS positive cells  RT (7)  LPS (8)  WBH (6) | One-way ANOVA  or  Kruskal-Wallis | Treatment _(RT, LPS, WBH)_  PVN: *F_(2,18)_* =12.44  LHab: *F_(2,18)_* =10.09 (KW)  CEA: *F_(2,18)_* =10.01 (KW)  PVT: *F_(2,18)_* =13.12  MPO: *F_(2,18)_* 13.35 (KW)  DMH: *F_(2,18)_* =22.23  LSV: *F_(2,18)_* =15.12 | p=0.0004  p=0.0029  p=0.0011  p=0.0003  p<0.0001  p<0.0001  p<0.0001 | Bonferroni or Dunn post-hoc  *(parametric or non-parametric)*  WBH vs RT, p = 0.0017  WBH vs RT, p = 0.0132  LPS vs RT, p = 0.0044  WBH vs RT, p = 0.0002  WBH vs RT, p = 0.0009  WBH vs RT, p <0.0001  WBH vs RT, p = 0.0003 |
| 4A | C57 mice  Glucose levels 4h  RT (8)  LPS (8)  WBH (10) | One-way ANOVA | Treatment _(RT, LPS, WBH)_  *F_(2,23)_* = 11.89 | p=0.0003 | Bonferroni post-hoc  RT vs LPS p=0.0002  RT vs WBH p=0.0094 |
| 4B | C57 mice  Glucose levels time-course  RT (8)  LPS (8)  WBH (8) | 3 x 6  Repeated measures two-way ANOVA | Treatment_(RT, LPS, WBH)_  *F_(2,21)_* =6.597  Time _( -1, 0.66, 2, 4, 7, 24 hrs)_  *F_(5,120)_* = 15.14  Treatment x Time  *F_(10, 120)_* = 1.904 | p=0.0060  p<0.0001  p=0.0530 | Bonferroni post-hoc  RT vs LPS (240min) p=0.0104  RT vs WBH (240min) p=0.0359  RT vs LPS (420min) p=0.0037  RT vs WBH (420min) p=0.0017 |
| 4C | C57 mice  Hypothalamic transcripts 4h  RT (4)  LPS (4)  WBH (5) | One-way ANOVA | Treatment_(RT, LPS, WBH)_  *Igf F_(2,10)_* =12.78  *Oxt F_(2,10)_* =0.6864  *Avp F_(2,10)_* =0.2099 | p=0.0018  p=0.5256  p=0.8141 | Bonferroni post-hoc  RT vs LPS p=0.054  RT vs WBH p=0.0015 |
| 5A | C57 mice  Activity Open field  RT (12)  LPS (8)  WBH (12) | 3 x 3  Repeated measures two-way ANOVA | Mean Speed  Treatment_(RT, LPS, WBH)_  *F_(2,29)_* =4.121  Time_(Baseline,5h, 24h)_  *F_(2,58)_* =9.898  Treatment x Time  *F_(4,58)_* =1.237  Immobile time  Treatment_(RT, LPS, WBH)_  *F_(2,29)_* =5.555  Time_(Baseline,5h, 24h)_  *F_(2,58)_* =19.74  Treatment x Time  *F_(4,58)_* =5.815  Rotations  Treatment_(RT, LPS, WBH)_  *F_(2,29)_* =4.832  Time_(Baseline,5h, 24h)_  *F_(2,58)_* =17.67  Treatment x Time  *F_(4,58)_* =1.274 | p=0.0266  p=0.0002  p=0.3052  p=0.0091  p<0.0001  p=0.0005  p=0.0154  p<0.0001  p=0.2905 | Bonferroni:  5h RT vs LPS p= 0.0206  5h WBH vs LPS p=0.0080  5h RT vs LPS p<0.0001  5h WBH vs LPS p<0.0001  5h RT vs LPS p=0.0219  5h WBH vs LPS p=0.0105 |
| 5B | C57 mice  Spontaneous behv.  RT (12)  LPS (8)  WBH (12) | 3 x 3  Repeated measures two-way ANOVA | Marble burying  Treatment_(RT, LPS, WBH)_  *F_(2,29)_* =4.184  Time_(Baseline,5h, 24h)_  *F_(2,58)_* =2.773  Treatment x Time  *F_(4,58)_* =3.005 | p=0.0253  p=0.0708  p=0.0254 | Bonferroni:  5h RT vs LPS p<0.0001  5h WBH vs LPS p=0.0051 |
| 5C | C57 mice  Social interaction  RT (12)  LPS (8)  WBH (12) | 3 x 3  Repeated measures two-way ANOVA | Time spent sniffing  Treatment_(RT, LPS, WBH)_  *F_(2,29)_* =0.0060  Time_(Baseline,5h, 24h)_  *F_(2,55)_* =7.334  Treatment x Time  *F_(4,55)_* =0.0024  Frequency sniffs  Treatment_(RT, LPS, WBH)_  *F_(2,29)_* =0.6822  Time_(Baseline,5h, 24h)_  *F_(2,58)_* =1.338  Treatment x Time  *F_(4,58)_* =0.3545 | p=0.9414  p=0.0062  p=0.9988  p=0.5083  p=0.2672  p=0.8402 |  |
| 6A | C57 (24) vs C58 (24)  Temperature | Unpaired t-test  Two-tailed | Temperature  t=4.139; df=46 | p=0.0001 |  |
| 6B | Spontaneous  behaviouur | Unpaired t-test  Two-tailed  Mann-Whitney | Burrowing 2h  t=4.139; df=46  Burrowing o.n.  U=158  Marble burying 20min  U=107 | p=0.0273  p=0.0041  p=0.0012 |  |
| 6C | Repetitive.  behaviour | Mann-Whitney Two-tailed | Backflips  U=0  Upright scrabbles  U=0 | p<0.0001  p<0.0001 |  |
| 6D | Activity, Open field | Unpaired t-test  Two-tailed  or  Mann-Whitney | Speed  t=5.940; df=46  Rotations  U=83  Time rearing  t=2.036; df=46  Distance  t=5.946; df=46 | p<0.0001  p<0.0001  p=0.0475  p<0.0001 |  |
| 6E | Social behaviour | Mann-Whitney Two-tailed | Frequency Sniffs  U=167  Time spent sniffing  U=153 | p=0.0196  p=0.0078 |  |
| 6F | C57 vs C58  Temperature timeline  C57 RT (12)  C57 WBH (12)  C58 RT (12)  C58 WBH (12) | 2x13  Repeated measures two-way ANOVA | C57  Treatment_(RT, WBH)_  *F_(1,22)_* =240.1  Time _(0-240mins)_  *F_(12,264)_* = 13.56  Treatment x Time  *F_(12,264)_* = 28.53  C58  Treatment_(RT, WBH)_  *F_(1,23)_* =139.2  Time _(0-240mins)_  *F_(12,276)_* = 8.826  Treatment x Time  *F_(12,276)_* = 7.625 | p<0.0001  p<0.0001  p<0.0001  p<0.0001  p<0.0001  p<0.0001 |  |
| 6G | C58 mice  WBH immediate effects at 5h  C58 RT (11)  C58 WBH (12) | Mann-Whitney Two-tailed  or  unpaired t-test  Two-tailed | Backflips  U=23  Upright scrabbles  U=12.5  Distance  t=0.4473  Frequency sniffs  U=21  Time spent sniffing  U=31.50 | p=0.0145  p=0.0077  p=0.6676  p=0.0044  p=0.0331 |  |
| 7A | C58 mice repetitive behaviours  C58 RT (10)  C58 WBH (12) | 2x3  Repeated measures two-way ANOVA | Upright Scr. time course  Treatment_(RT, WBH)_  *F_(1,20)_* =4.75  Time_(0, 24h, 48h)_  *F_(2,40)_* =7.78  Treatment x Time  *F_(2,40)_* =0.95  Backflips time course  Treatment_(RT, WBH)_  *F_(1,20)_* =5.99  Time_(0, 24h, 48h)_  *F_(2,40)_* =0.81  Treatment x Time  *F_(2,40)_* =6.83 | p=0.0413  p=0.0017  p=0.3936  p=0.0237  p=0.4510  p=0.0028 | Fisher’s LSD RT 24h vs WBH 24h  p=0.0329  Fisher’s LSD RT 24h vs WBH 24h  p=0.0017  RT 48h vs WBH 48h  p=0.0069 |
| 7B | C58 mice locomotor activity  C58 RT (11)  C58 WBH (12) | 2x2  Repeated measures two-way ANOVA | Mean Speed  Treatment_(RT, WBH)_  *F_(1,21)_* =0.698  Time_(0, 24)_  *F_(1,21)_* =0.0674  Treatment x Time  *F_(1,21)_* =2.86  Rotations  Treatment_(RT, WBH)_  *F_(1,21)_* =0.6530  Time_(0, 24)_  *F_(1,21)_* =0.0568  Treatment x Time  *F_(1,21)_* =1.883 | p=0.4129  p=0.7977  p=1.054  p=0.4821  p=0.8139  p=0.1845 |  |
| 7C | C58 social interaction at 24h  C58 RT (11)  C58 WBH (12) | Mann Whitney two-tailed | Frequency Sniffs 24h  U=48.50  Time spent sniffing  U=65 | p=0.1824  p=0.9640 |  |
| 8A | C57 vs Shank3b  Temperature  C57 (23)  Shank3b (25) | Mann Whitney two-tailed | Temperature  U=174.5 | p=0.0467 |  |
| 8B | Spontaneous  behaviour | Unpaired t-test  Two-tailed | Marble burying 20min  t=1.096; df=46 | p=0.2787 |  |
| 8C | Repetitive behaviour | Mann Whitney two-tailed | Grooming  U=181 | p=0.0278 |  |
| 8D | Muscular coordination. | Mann Whitney two-tailed | Horizontal Bar  U=105  Success  U= 121 | p<0.0001  p<0.0001 |  |
| 8E | Risk assessment and anxiety | Unpaired t-test  Two-tailed  or  Mann Whitney | Latency  U= 134  Only neck  t=3.607; df=46  Two paws  t=2.771; df=46  Total risk assessment  U= 116  Time Open Zone  t=3.336; df=46 | p=0.0012  p=0.0008  p=0.0080  p=0.0002  p=0.0017 |  |
| 8F | Activity Open field | Unpaired t-test  Two-tailed | Speed  t=0.6930; df=46  Rotations  t=0.5378; df=46  Time rearing  t=1.1417; df=46  Distance  t=0.6759; df=46 | p=0.4918  p=0.5933  p=0.1631  p=0.5025 |  |
| 8G | Social interaction | Mann Whitney  Two-tailed | Frequency sniffs  U=253  Time spent sniffing  U=268 | p=0.4864  p=0.6857 |  |
| 9A | C57 vs Shank3b  Temperature timeline  C57 RT (14)  C57 WBH (16)  Shank3b RT (17)  Shank3b WBH (18) | 2x13  Repeated measures two-way ANOVA | C57  Treatment_(RT, WBH)_  *F_(1,28)_* =143.1  Time _(0-240mins)_  *F_(12,324)_* = 10.99  Treatment x Time  *F_(12,324)_* = 14.85  Shank3b  Treatment_(RT, WBH)_  *F_(1,33)_* =93.27  Time _(0-240mins)_  *F_(12,396)_* = 10.99  Treatment x Time  *F_(12,396)_* = 17.69 | p<0.0001  p<0.0001  p<0.0001  p<0.0001  p<0.0001  p<0.0001 |  |
| 9B | Shank3b mice  Grooming time  Shank3b RT (15)  Shank3b WBH (15) | 2x3  Repeated measures two-way ANOVA  Mann Whitney  Two-tailed | Grooming baseline, 24, 48h  Treatment_(RT, WBH)_  *F_(1,28)_* =5.46  Time _(Baseline-48h)_  *F_(2,56)_* = 13.52  Treatment x Time  *F_(2,56)_* = 5.25  Grooming 24h  U=24 | p=0.0268  p=0.0081  p=0.0081  p=0.0002 | Bonferroni:  RT 24h vs WBH 24h p=0.0014 |
| 9C | Shank3b mice  Social Interaction  Shank3b RT (15)  Shank3b WBH (16) | Kruskal-Wallis non-parametric analysis | Frequency sniffs  Time spent sniffing | p=0.0336  p=0.0141 | Dunn’s Test  WBH 5h vs RT 5h p=0.0062  WBH 5h vs RT 5h p=0.0054 |
| 9D | Shank3b mice  Elevated Zero Maze  Shank3b RT (11)  Shank3b WBH (14) | Two-way ANOVA  Mann Whitney  Two-tailed  Unpaired t-test  Two-tailed | Latency timeline Shank3b  Treatment_(RT, WBH)_  *F_(1,23)_* =0.3635  Time _(Baseline-48h)_  *F_(3,69)_* = 12.94  Treatment x Time  *F_(3,69)_* = 0.2289  Latency 5h  U=37  Time Open Zone at 5h  t=1.280 | p=0.5525  p=0.0001  p=0.8760  p=0.1402  p=0.7575 |  |

***Transcription of Heat Shock Protein genes at 4 and 24h after WBH or LPS***


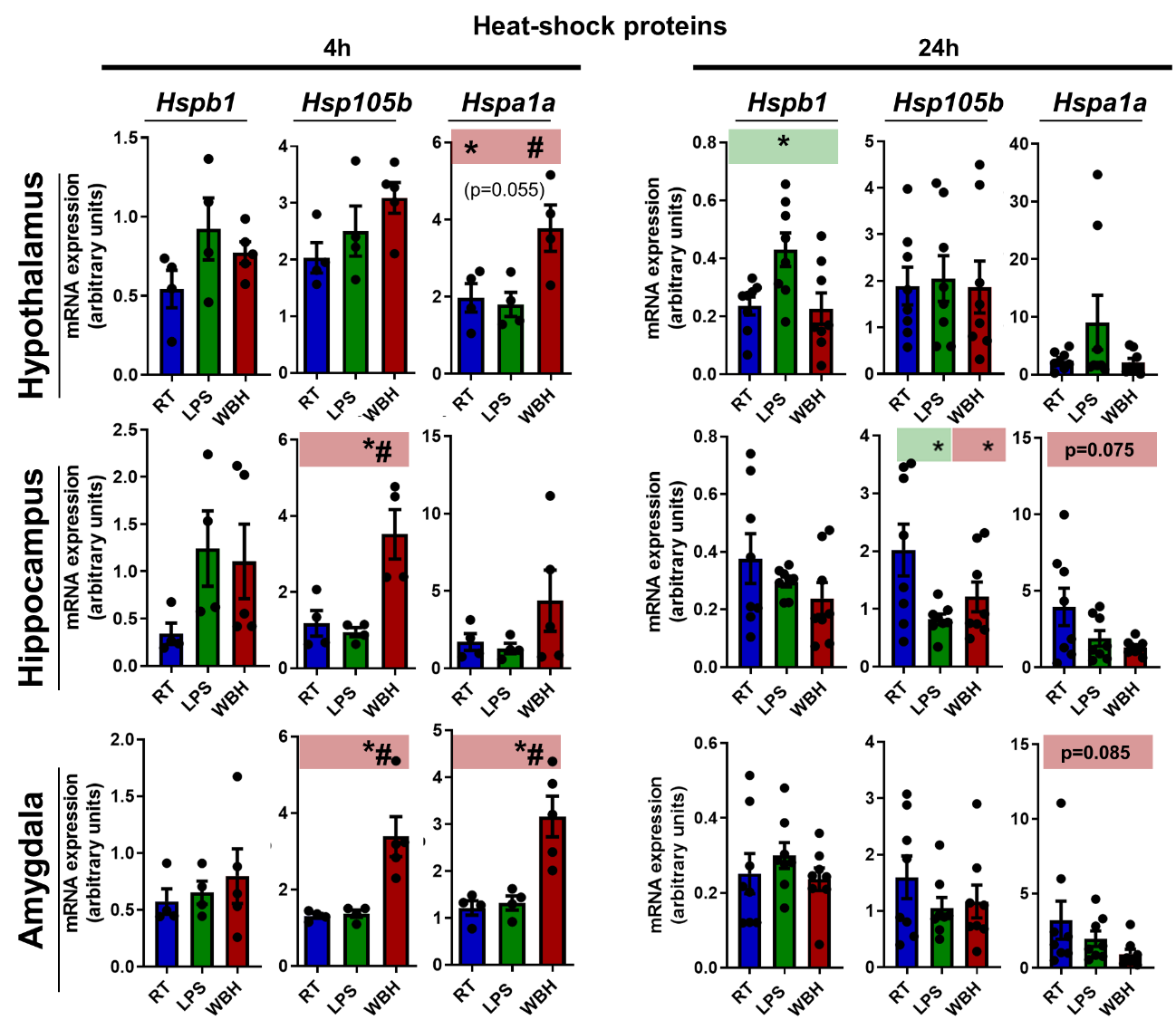


**Additional file Fig. S1**: Level of expression of mRNA for heat shock proteins in brain homogenates of hypothalamus, hippocampus, and amygdala at 4h (left, RT n=4; LPS n=4; WBH n=6) and 24h (right; n=8) after LPS or WBH treatment. Data are shown as mean ± SEM, with each data point representing one animal. Data analysed by one-way ANOVA followed Bonferroni’s test. * vs. RT; # vs. LPS. Red background around statistical symbols means general effect of WBH, whereas green means LPS effect. Abbreviations: RT, room temperature; LPS, intraperitoneally injected LPS, 250µg/kg; WBH, whole-body hyperthermia.

Heat shock proteins (HSPs) are chaperone proteins that minimise protein denaturation and aggregation under heat shock (42-45 °C) and febrile-temperatures (38–41 °C), among other stressors [34]. *De novo* transcription of HSPs was analysed at 4 and 24 hours in brain homogenates of the hypothalamus, hippocampus, and amygdala. Although quite variable, WBH did trigger increased expression of Hspa1a and Hsp105B in all three regions and these increases were significant in hippocampus and amygdala for Hsp105b (p=0.0038, p=0.0062, respectively) and in the hypothalamus and the amygdala for Hspa1a (p=0.0214, p=0.0017 respectively). None of these changes remained at 24 hours. Although there were some modest increases, neither WBH nor LPS significantly increased Hspb1 and indeed LPS did not significantly increase any HSP at 4 hours. Conversely, Hsp1b was increased in the hypothalamus 24 hours after LPS treatment (p=0.0341) and Hsp105b was suppressed at 24 hours in the hippocampus (p=0.0332). See suppl. table 2 for further statistical descriptions.

***LPS reduces spontaneous activity***

**
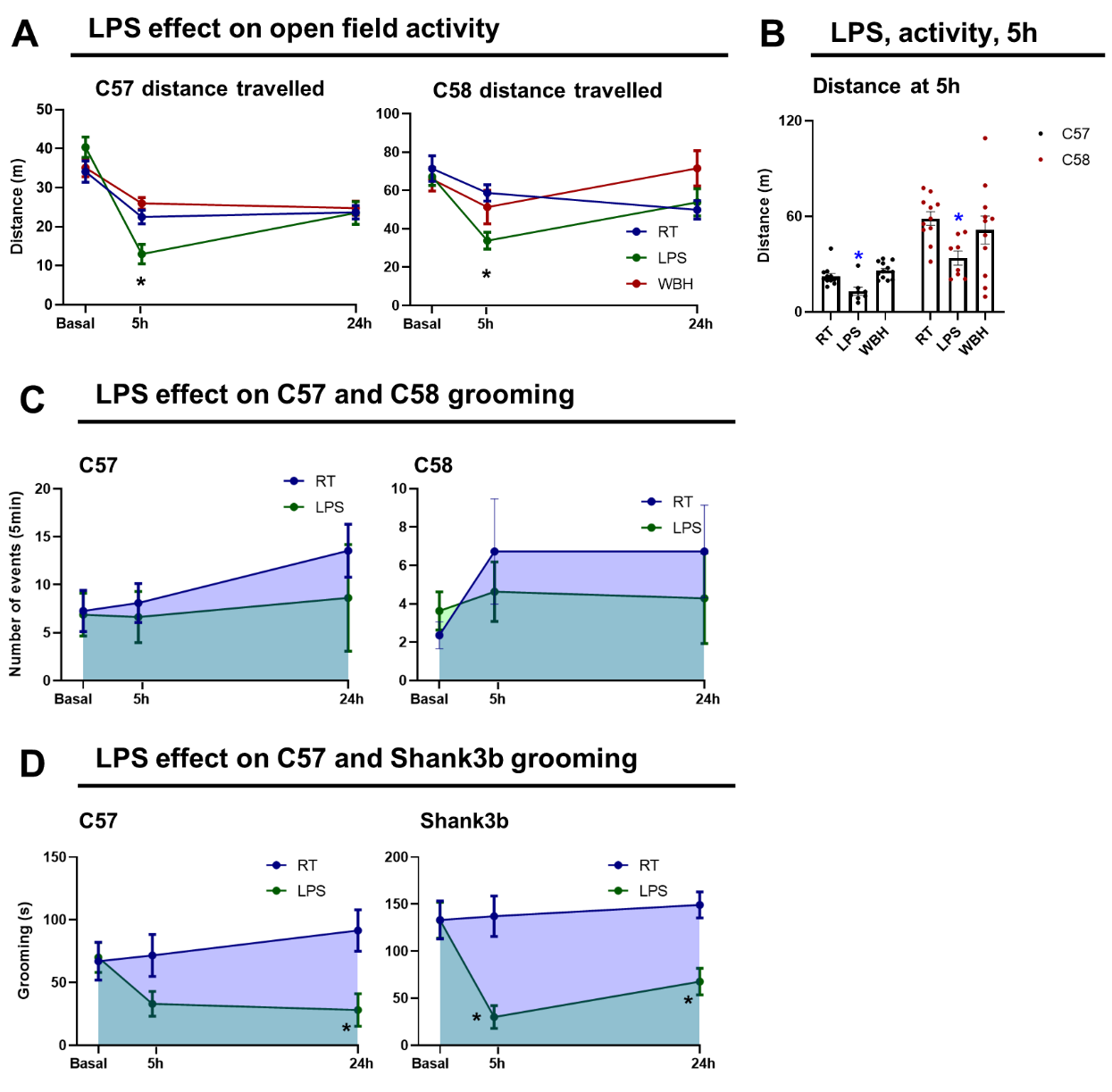
**

**Additional file, Fig. S2: Effects of LPS on general activity in C57, C58 and Shank3b mice.** (A) Distance travelled time course in C57 and C58 (C57: RT n=12; LPS n=8; WBH n=12 // C58: RT n=12; LPS n=8; WBH n=13). Data were analysed by repeated measures two-way ANOVA followed Bonferroni’s test. (B) Distance travelled at 5h, comparison of C57 and C58. Two-way ANOVA analysis followed by Bonferroni’s test. (C) Number of grooming events time course in C57 and C58 after LPS treatment. Repeated measures two-way ANOVA. (D) Time spent grooming time course in C57 and Shank3b after LPS treatment. Repeated measures two-way ANOVA. Data are shown as mean ± SEM. * vs. RT. Abbreviations: RT, room temperature; LPS, intraperitoneally injected LPS, 250µg/kg; WBH, whole-body hyperthermia.

The injection of LPS (250µg/kg; i.p.) led to a marked decrease in general activity as assessed by the distance travelled in the open field, which was transient and restored to baseline levels within 24 hours after injection in C57 and C58 mice (A). Two-way ANOVA repeated measures followed by Bonferroni post hoc test demonstrated that the activity significantly decreased in C57 (p=0.0230) and C58 (p=0.0028) at 5 hours after treatment, with no significant difference between the two strains. Notably, the decrease in distance travelled at 5 hours was particular to LPS treatment, as WBH did not cause any change in general activity (B). Importantly, LPS treatment did not affect the number of grooming events in C57 and C58 during the habituation period of the open field test (C). However, when the time spent grooming was assessed using a specific grooming test, repeated measures two-way ANOVA showed a significant reduction in grooming time in C57 mice at 24 hours (p=0.0226), whereas Shank3b animals showed significant reductions at both 5 hours (p=0.0007) and 24 hours (p=0.0015) after treatment (D).

***C57, C58 and Shank3b temperature comparison***


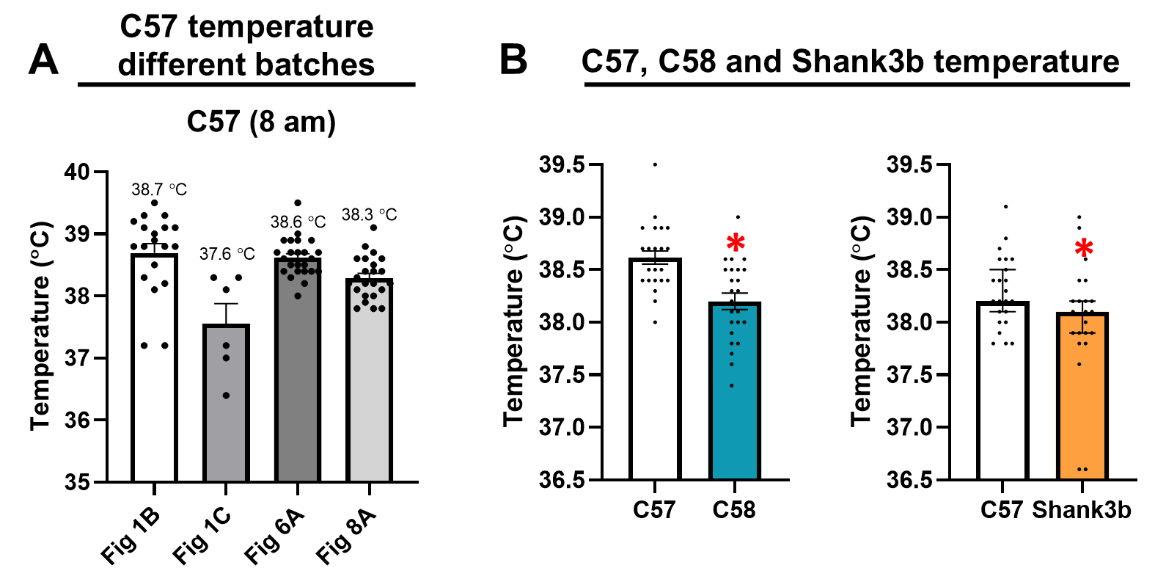


**Additional file Fig. S3: C57, C58 and Shank3b temperature comparison.** (A) C57 temperature comparison in all different experimental batches used. Data are presented as mean ± SEM or median ± IQR in the case of Shank3b temperature, with each data point representing one animal. * vs. C57, * vs. RT. Abbreviations: RT, room temperature; LPS, intraperitoneally injected LPS, 250µg/kg; WBH, whole-body hyperthermia.

The temperature of C57 mice remained relatively uniform throughout the experimental batches, as depicted in Suppl. Fig. 3A. However, the data from Figure 1C were significantly lower than that of the other batches (p=0.0012 vs Fig1B and p=0.0073 vs Fig6A). This difference can be attributed to the intracerebral thermocouple implantation procedure that was applied exclusively to the animals used to generate Figure 1C. This differential treatment resulted in a lower basal temperature for this subset of animals. In Supplemental Figure 3B, we compare two different C57 batches with their respective counterparts (C58 and Shank3b). Student t-test shows that C58 had a lower basal temperature than C57 (p=0.0001), while Shank3b also exhibited a significantly lower temperature (p=0.0467), as determined by Mann-Whitney pair-wise comparison.

***WBH increases the grooming number of events at 5h in all animals:***


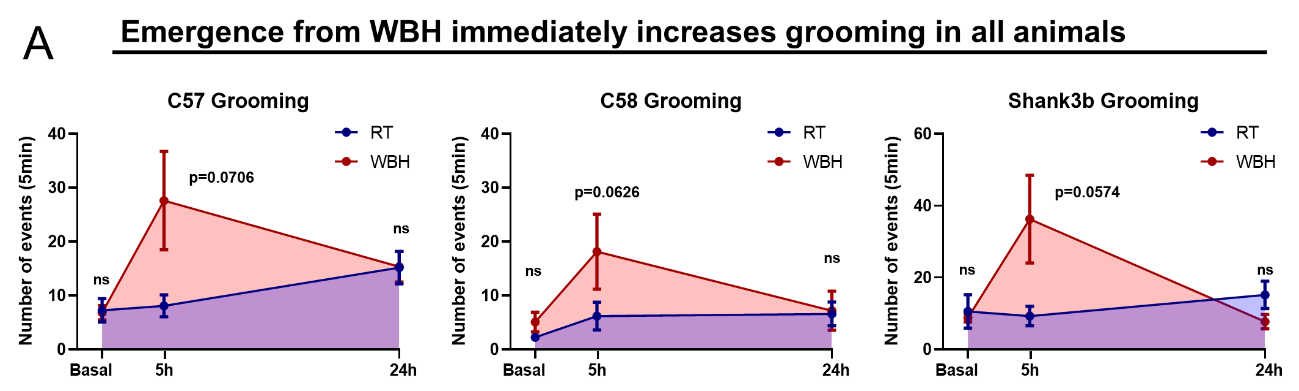


**Additional file Fig. S4. WBH increases grooming number of events.** Number of grooming events observed in the open field (5min), after removal from the heating protocol in C57, C58 and Shank3b in RT and WBH groups. Repeated measures two-way ANOVA followed by Bonferroni post hoc test showed a trend to be increased in C57 (p=0.0706); C58 (p=0.0626) and Shankb3 (p=0.0574). Data are shown as mean ± SEM. * vs. RT. Abbreviations: RT, room temperature; WBH, whole-body hyperthermia.

Our results indicate that after removing the animals from the heating chamber, all animals exhibited an increase in grooming behaviour that was transient and variable but had returned to baseline levels when measured at 24 hours. Specifically, repeated measures two-way ANOVA followed by Bonferroni post hoc tests showed a trend to be increased in C57 (p=0.0706); C58 (p=0.0626) and Shankb3 (p=0.0574).

***Shank3b complete behaviour battery tests post-WBH***


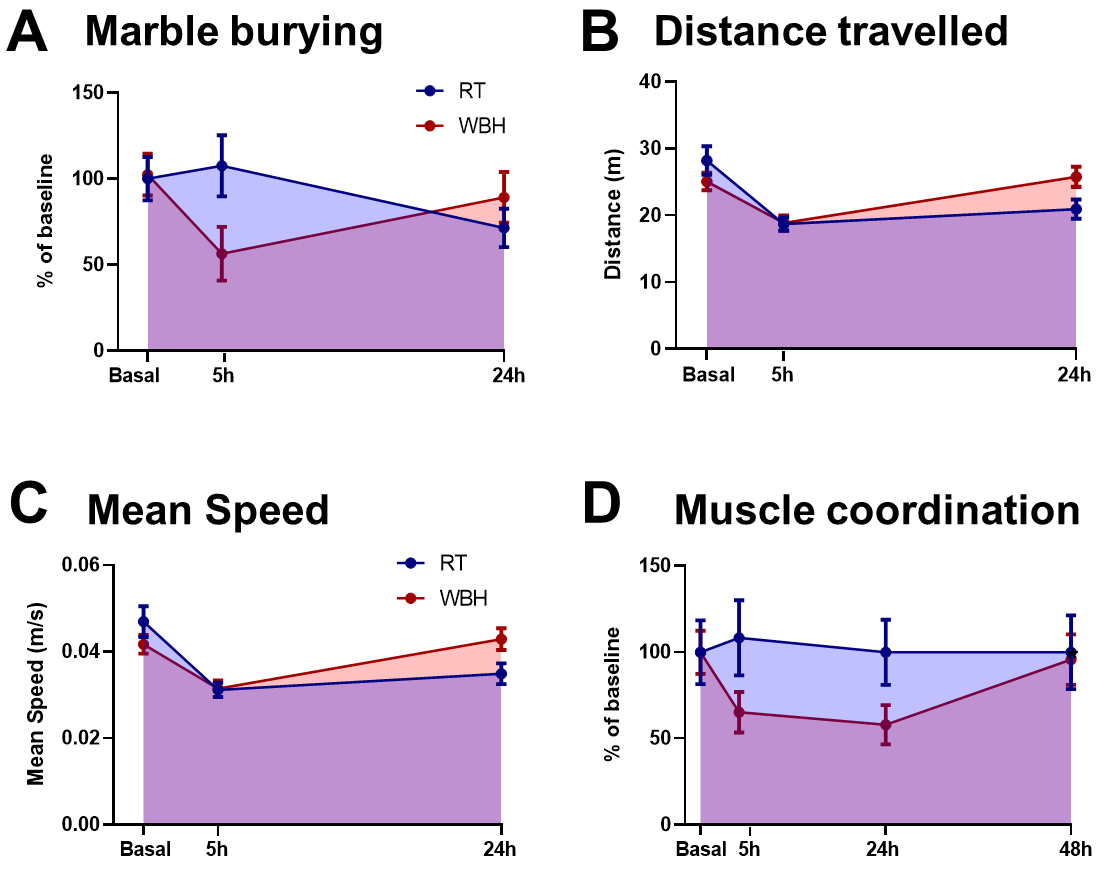


**Additional file Fig. S5. Shank3b battery of behavioural tests.** (A) Marble burying percentage of baseline time course. (B) Distance travelled (m) assessed during the open field test, time course. (C) Mean speed (m/s) assessed during the open field test, time course. (D) Muscle coordination on the horizontal bar test, as a percentage of baseline. All behaviours are compared in Shank3B mice following RT and WBH treatment. Data are shown as mean ± SEM. RT. Abbreviations: RT, room temperature; WBH, whole-body hyperthermia.

Shank3b mice were subjected to a complete battery of behavioural tests, as shown in Figure 8. Baseline characterization of the strain was performed (Fig 8), and some of those tests showing significant differences were represented in Figure 9 to assess the effects of WBH. All tests were conducted at each time point. Specifically, the time courses of spontaneous behaviour (A), general activity measured by distance travelled (B) and mean speed (C), and muscle/motor coordination on the horizontal bar (D) were analysed. Repeated measures two-way ANOVA analysis revealed that WBH treatment did not affect any of these behavioural measures.

***Additional file, table 2: Statistical analyses for data in the supplemental section***

| **Figure** | **Group (n)** | **Test used** | **Statistical values** | ***p* value** | **Post-hoc tests** |
| --- | --- | --- | --- | --- | --- |
| Suppl.1A  (left) | Distance travelled time course.  C57:  -RT (12)  -LPS (8)  -WBH (12) | Repeated measures two-way ANOVA | Treatment_(RT, LPS, WBH)_  *F_(2,29)_* =1.684  Time_(0-24h)_  *F_(2,46)_* =39.23  Treatment x Time  *F_(4,58)_* =4.078 | p=0.2033  p<0.0001  p=0.0056 | Bonferroni:  5h RT vs LPS p= 0.0230 |
| Suppl.1A  (right) | Distance travelled time course.  C58:  -RT (12)  -LPS (8)  -WBH (13) | Repeated measures two-way ANOVA | Treatment_(RT, LPS, WBH)_  *F_(2,27)_* =1.483  Time_(0-24h)_  *F_(2,46)_* =8.191  Treatment x Time  *F_(4,54)_* = 2.830 | p=0.2448  p=0.0009  p=0.0333 | Bonferroni:  5h RT vs LPS p= 0.0083 |
| Suppl. 1B | Distance travelled at 5h  C57 / C58:  -RT (12/12)  -LPS (8/8)  -WBH (12/13) | Two-way ANOVA | Treatment_(RT, LPS, WBH)_  *F_(2,56)_* =7.009  Strain_(C57, C58)_  *F_(1,56)_* =50.15  Treatment x Strain  *F_(2,56)_* = 1.391 | p=0.0019  p<0.0001  p=0.2572 | Bonferroni:  C57 RT vs LPS p= 0.0050  C58 RT vs LPS p= 0.0492 |
| Suppl.1C  (left) | Number grooming events time course.  C57:  -RT (12)  -LPS (8) | Repeated measures two-way ANOVA | Treatment_(RT, LPS)_  *F_(1,53)_* =0.8630  Time_(0-24h)_  *F_(2,46)_* =1.129  Treatment x Time  *F_(2,53)_* =0.3165 | p=0.3571  p=0.3232  p=0.7300 |  |
| Suppl.1C  (right) | Number grooming events time course.  C58:  -RT (12)  -LPS (8) | Repeated measures two-way ANOVA | Treatment_(RT, LPS)_  *F_(1,50)_* =0.4159  Time_(0-24h)_  *F_(2,46)_* =1.062  Treatment x Time  *F_(2,50)_* =0.4901 | p=0.3398  p=0.5219  p=0.6154 |  |
| Suppl.2 | C57 mice  Heat-shock prot.  Hypothalamus 4h  RT (4)  LPS (4)  WBH (5) | One-way ANOVA | Treatment_(RT, LPS, WBH)_  *Hspb1 F_(2,10)_* =2.022  *Hsp105b F_(2,10)_* =2.681  *Hspa1a F_(2,9)_* =6.069 | p=0.1830  p=0.1169  p=0.0214 | Bonferroni:  LPS vs WBH p=0.0355 |
| Suppl.2 | C57 mice  Heat-shock prot.  Hippocampus 4h  RT (4)  LPS (4)  WBH (5) | One-way ANOVA | Treatment_(RT, LPS, WBH)_  *Hspb1 F_(2,10)_* =1.864  *Hsp105b F_(2,9)_* =11.01  *Hspa1a F_(2,10)_* =1.551 | p=0.2052  p=0.0038  p=0.2591 | Bonferroni:  RT vs WBH p=0.0116  LPS vs WBH p=0.0065 |
| Suppl.2 | C57 mice  Heat-shock prot.  Amygdala 4h  RT (4)  LPS (4)  WBH (5) | One-way ANOVA | Treatment_(RT, LPS, WBH)_  *Hspb1 F_(2,10)_* =0.4088  *Hsp105b F_(2,10)_* =11.47  *Hspa1a F_(2,10)_* =12.87 | p=0.6751  p=0.0062  p=0.0017 | Bonferroni:  RT vs WBH p=0.0062  LPS vs WBH p=0.0074  RT vs WBH p=0.0038  LPS vs WBH p=0.0056 |
| Suppl.2 | C57 mice  Heat-shock prot.  Hypothalamus 24h  RT (8)  LPS (8)  WBH (8) | One-way ANOVA | Treatment_(RT, LPS, WBH)_  *Hspb1 F_(2,21)_* =5.396  *Hsp105b F_(2,21)_* =0.04098  *Hspa1a F_(2,21)_* =12.87 | p=0.0128  p=0.9599  p=1.969 | Bonferroni:  LPS vs RT p=0.0341  LPS vs WBH p=0.0250 |
| Suppl.2 | C57 mice  Heat-shock prot.  Hippocampus 24h  RT (8)  LPS (8)  WBH (8) | One-way ANOVA | Treatment_(RT, LPS, WBH)_  *Hspb1 F_(2,21)_* =1.351  *Hsp105b F_(2,21)_* =4.043  *Hspa1a F_(2,21)_* =3.206 | p=0.2805  p=0.0327  p=0.0609 | Bonferroni:  LPS vs RT p=0.0332  RT vs WBH p=0.075 |
| Suppl. 2 | C57 mice  Heat-shock prot.  Amygdala 24h  RT (8)  LPS (8)  WBH (8) | One-way ANOVA  Kruskal Wallis | Treatment_(RT, LPS, WBH)_  *Hspb1 F_(2,21)_* =0.6586  *Hsp105b F_(2,21)_* =0.9397  *Hspa1a F_(2,21)_* =5.345 | p=0.5280  p=0.4066  p=0.0691 | Bonferroni:  RT vs WBH p=0.085 |
| Suppl. 3A | C57 temperature  Fig1B (19)  Fig1C (6)  Fig6A (24)  Fig8A (23) | Kruskal Wallis | Temperature  F*_(4,72)_*=21.60 | p<0.0001 | MannWhitney  1B vs 1C, p=0.0012  6A vs 1C, p=0.0073 |
| Suppl. 3B  (left) | All strains temperature  C57, left (24)  C58 (24) | Unpaired t-test  Two-tailed | Temperature  t=4.139; df=46 | p=0.0001 |  |
| Suppl. 3B  (right) | All strains temperature  C57, right (23)  Shank3b (23) | Mann-Whitney | Temperature  U=174.5 | p=0.0467 |  |
| Suppl.4  (C57) | Number grooming events  C57 RT (12)  C57 WBH (12) | Repeated measures two-way ANOVA | Treatment_(RT, WBH)_  *F_(1,21)_* =3.510  Time_(0-24h)_  *F_(2,31)_* =4.364  Treatment x Time  *F_(2,41)_* =4.328 | p=0.0750  p=0.0306  p=0.0197 | Bonferroni:  Trend at 5h, p=0.0706 |
| Suppl.4  (C58) | Number grooming events  C58 RT (12)  C58 WBH (13) | Repeated measures two-way ANOVA | Treatment_(RT, WBH)_  *F_(1,67)_* =2.833  Time_(0-24h)_  *F_(2,41)_* =2.661  Treatment x Time  *F_(2,67)_* =1.323 | p=0.0790  p=0.1032  p=0.2733 | Bonferroni:  Trend at 5h, p=0.0626 |
| Suppl.4  (Shank3b) | Number grooming events  Shank3b RT (16)  Shank3b WBH (17) | Repeated measures two-way ANOVA | Treatment_(RT, WBH)_  *F_(1,31)_* =1.227  Time_(0-24h)_  *F_(2,41)_* =3.235  Treatment x Time  *F_(2,61)_* =5.215 | p=0.2766  p=0.0688  p=0.0081 | Bonferroni:  Trend at 5h, p=0.0574 |
| Suppl.5A | Marble burying  Shank3b RT (16)  Shank3b WBH (17) | Repeated measures two-way ANOVA | Treatment_(RT, WBH)_  *F_(1,37)_* =0.7756  Time_(0-24h)_  *F_(2,48)_* =1.377  Treatment x Time  *F_(2,37)_* =3.295 | p=0.3842  p=0.2622  p=0.0482 |  |
| Suppl.5B | Distance travelled  Shank3b RT (16)  Shank3b WBH (17) | Repeated measures two-way ANOVA | Treatment_(RT, WBH)_  *F_(1,33)_* =0.1636  Time_(0-24h)_  *F_(2,66)_* =21.14  Treatment x Time  *F_(2,66)_* =5.478 | p=0.6885  p<0.0001  p=0.0063 |  |
| Suppl.5C | Mean Speed  Shank3b RT (16)  Shank3b WBH (17) | Repeated measures two-way ANOVA | Treatment_(RT, WBH)_  *F_(1,33)_* =0.1626  Time_(0-24h)_  *F_(2,66)_* =20.89  Treatment x Time  *F_(2,66)_* =5.424 | p=0.6893  p<0.0001  p=0.0066 |  |
| Suppl.5D | Horizontal bar  Shank3b RT (16)  Shank3b WBH (17) | Repeated measures two-way ANOVA | Treatment_(RT, WBH)_  *F_(1,33)_* =1.555  Time_(0-48h)_  *F_(3,33)_* =1.248  Treatment x Time  *F_(3,99)_* =1.768 | p=0.2212  p=0.2961  p=0.1582 |  |

Abbreviations, supplemental data:

Hsp105B Heat shock protein 105B

Hsp1b Heat shock protein family B [small] member 1

Hspa1a Heat shock 70 kDa protein 1

HSPs Heat shock proteins

LPS intraperitoneally injected LPS, 250µg/kg

RT Room temperature

WBH Whole body hyperthermia
